# Increased environmental microbial diversity reduces the disease risk of a mosquitocidal pathogen

**DOI:** 10.1128/mbio.02726-23

**Published:** 2023-12-06

**Authors:** Zhiwei Kang, Vincent G. Martinson, Yin Wang, Kerri L. Coon, Luca Valzania, Michael R. Strand

**Affiliations:** 1 Hebei University, College of Life Sciences, Baoding, Hebei, China; 2 Department of Entomology, University of Georgia, Athens, Georgia, USA; 3 Department of Biology, University of New Mexico, Albuquerque, New Mexico, USA; 4 Department of Bacteriology, University of Wisconsin-Madison, Madison, Wisconsin, USA; 5 Institut Curie, Paris, France; National Institute of Allergy and Infectious Diseases, Bethesda, Maryland, USA

**Keywords:** mosquito, *Chromobacterium*, microbiota, virulence factor

## Abstract

Increased diversity in host-associated microbial communities often benefits animal health by provisioning nutrients and protecting against pathogens. In contrast, whether the diversity of environmental microbial communities can also benefit animal health is much less studied. In aquatic environments, water enables environmental microbes to more directly interact with pathogens and animals than in terrestrial environments, which also suggests the potential for community diversity to benefit animal health. In this study, we addressed this question by identifying a strain of *Chromobacterium haemolyticum* named Rain 0013 (*Ch_*R13) that is a virulent pathogen of mosquito larvae, which live in aquatic habitats. Comparative genomic and functional data suggested many *Chromobacterium* are both mosquitocidal and opportunistic pathogens of other animals due to conservation of virulence factors that include a broad-spectrum toxin (cyanide). However, we also identified a strongly negative correlation between diversity of the environmental microbiota and disease severity due to *Ch_*R13 needing to achieve a threshold density to kill mosquito larvae. Experiments using defined (gnotobiotic) microbial communities further suggested certain environmental community members reduced *Ch_*R13 densities more than others. Altogether, our results indicate *Ch_*R13 and other *Chromobacterium* have the potential to broadly cause disease but the environmental communities of microbes in aquatic habitats attenuate disease risk.

**IMPORTANCE:**

The host-specific microbiotas of animals can both reduce and increase disease risks from pathogens. In contrast, how environmental microbial communities affect pathogens is largely unexplored. Aquatic habitats are of interest because water enables environmental microbes to readily interact with animal pathogens. Here, we focused on mosquitoes, which are important disease vectors as terrestrial adults but are strictly aquatic as larvae. We identified a pathogen of mosquito larvae from the field as a strain of *Chromobacterium haemolyticum*. Comparative genomic analyses and functional assays indicate this strain and other *Chromobacterium* are mosquitocidal but are also opportunistic pathogens of other animals. We also identify a critical role for diversity of the environmental microbiota in disease risk. Our study characterizes both the virulence mechanisms of a pathogen and the role of the environmental microbiota in disease risk to an aquatic animal of significant importance to human health.

## INTRODUCTION

Many traits affect the virulence and transmissibility of animal pathogens including host-specific microbiotas that can either confer protection or increase disease risks (1–4). Less studied is how environmental microbial communities affect animal pathogens. Aquatic environments are especially interesting in this regard because the physiochemical properties of water enable commensal microbes and pathogens to interact with one another and different animals, while also transporting broad-spectrum toxins some microbes produce that can also adversely affect animal health (5, 6). Microbes in aquatic habitats additionally serve as the primary source of the host-specific microbiotas that assemble in different aquatic animal species (7, 8).

Among the simplest but most abundant freshwater habitats are small containers that fill with rainwater (9–11). Plant detritus, animal detritus, and soluble nutrients form the base of the food web, while microbes form a community that consists of one or more decomposer trophic levels (12, 13). An estimated 40% of all mosquito species develops in container habitats where terrestrial adult females lay eggs and aquatic larvae function as top-level consumers (12). Several of these species are medically important because adult females must blood feed on humans to produce eggs, which can result in transmission of diseases like malaria, Dengue fever and filariasis (14).

Microbial communities in container habitats include fungi, protists, and algae but predominantly consist of bacteria (15, 16). At the species level, bacteria community composition also commonly varies within and between geographic locations (17–26). Unlike microbial communities in terrestrial ecosystems, the microbes in aquatic container habitats constantly cycle through the digestive tract of mosquito larvae as they feed (27, 28). Combined with having to molt to grow, larvae thus do not establish stable, resident gut microbiotas. Under most culture conditions, microbes cycling through the digestive tract provision essential nutrients like B vitamins that larvae require for the development into adults (19, 24, 29–32). Conventional (CN) mosquito cultures reared in the laboratory likewise host communities of environmental microbes that are required for development. Previous work has (i) cultivated several of the commensal bacteria from field and laboratory cultures and (ii) developed methods for producing axenic (AX) cultures with no microbes or gnotobiotic (GN) cultures with defined communities of microbes (19, 29–33). Most known pathogens of mosquito larvae are also bacteria. Species like *Bacillus thuringiensis israelensis* and *Lysinibacillus sphaericus* are used worldwide as larvicides (34, 35), while *Chromobacterium* is a genus of Gram-negative Betaproteobacteria that includes several isolates that kill mosquitoes (36–40).

This study arose from monitoring container habitats for mosquitoes in the genera *Aedes* and *Culex* in Southeastern United States that preferentially reside in urban landscapes and are human disease vectors (41). Water from one container lacking mosquitoes killed mosquito larvae in laboratory bioassays, which prompted two downstream objectives. The first was to identify the causative agent, which was a strain of *Chromobacterium haemolyticum* we named Rain 0013 (*Ch_*R13). A genus-wide phylogeny, comparative genomic analysis, and functional assays suggested most if not all bacteria in the *C. haemolyticum* group are opportunistic pathogens with wide host ranges due to each producing a broad-spectrum toxin (cyanide) and other virulence factors. The second objective took advantage of *Ch_*R13 and the simplicity of container habitats relative to most aquatic habitats to address how environmental microbial communities affect pathogen invasion and disease severity. Results showed a negative correlation between diversity of the container microbiota and disease severity, which was due to *Ch_*R13 not reaching a threshold density to kill mosquito larvae. Collectively, our results increase the understanding of *Chromobacterium* spp. as mosquito pathogens while providing new insights on how environmental communities of microbes affect the disease risk in aquatic animals.

## RESULTS

### *Ch_*R13 is a mosquitocidal strain of *Chromobacterium haemolyticum*

We sampled several container habitats in Athens, GA, USA, from May to October 2017 for *Aedes* and *Culex* larvae. A container named “Rain” was a bucket located 2 km away from the laboratory that had few or no mosquito larvae over several collection dates, whereas other containers had larvae during the same period. Adding first instar *Aedes aegypti*, *Aedes albopictus*, or *Culex quinquefasciatus* from our CN laboratory cultures to water from the “Rain” bucket resulted in most larvae dying in 3 days (Fig. S1). In contrast, autoclaving or passing “Rain” water through an 0.2-µm filter before use resulted in little or no mortality, which suggested activity was due to a biological agent (Fig. S1). A glycerol stock prepared from “Rain” water was streaked onto several types of solid medium including low nutrient 1/10 dilution 869 agar plates. Individual colonies were then inoculated into sterile water to which *A. aegypti*, *A. albopictus*, or *C. quinquefasciatus* first instars plus rearing diet were added. Larvae died in 1–3 days when water was inoculated with colonies from low-nutrient 869 plates that shared a common morphology (white, smooth margin) (Fig. S1). Little or no larval mortality was observed for any of the other culturable microbes we tested. The 16S rRNA gene from the colonies exhibiting larvicidal activity was assigned to the genus *Chromobacterium*.

**Fig 1:**
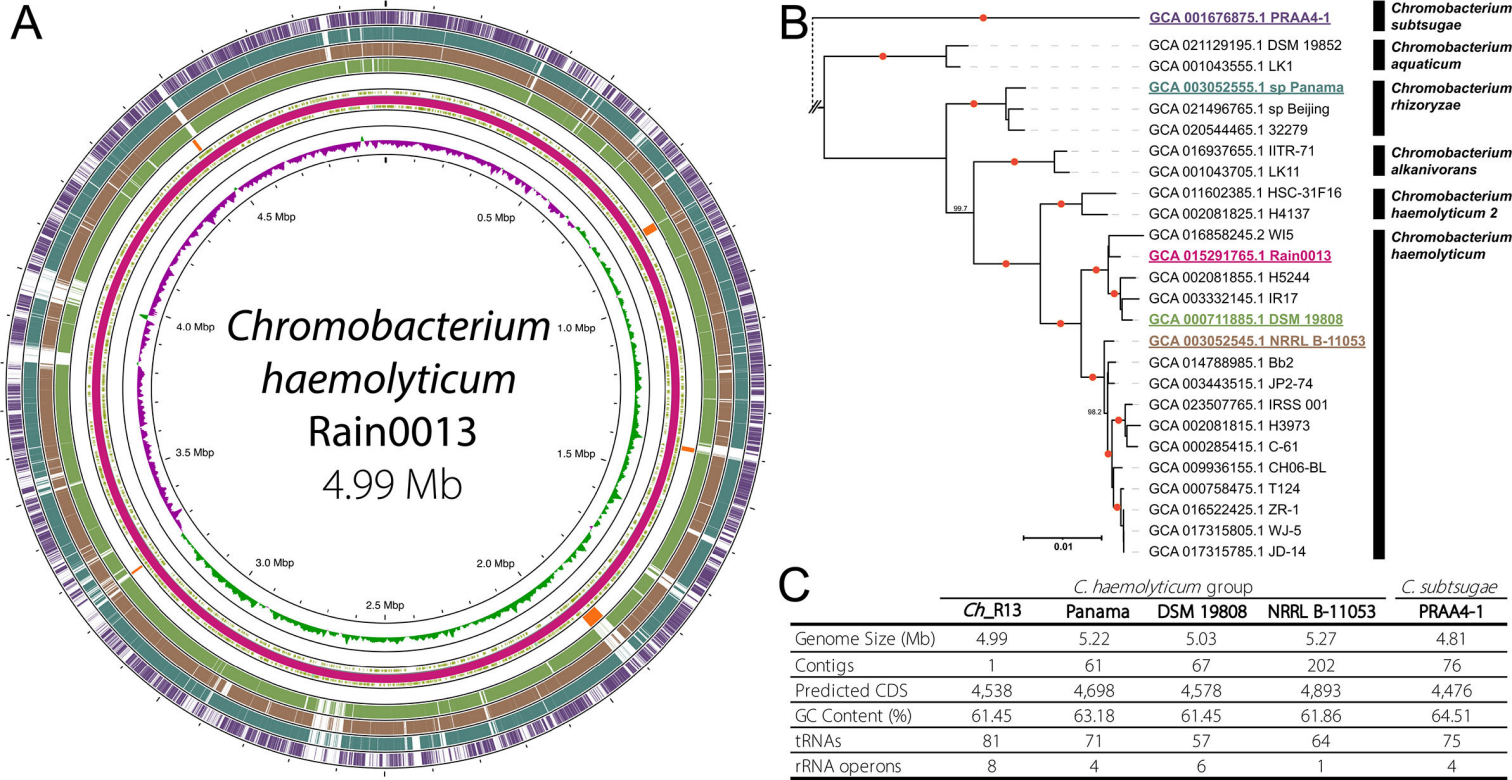
Genome assembly and phylogenetic analysis of *Chromobacterium haemolyticum* Rain0013 (*Ch_*R13). (**A**) Circular genome map for *Ch_*R13 (pink ring) showing predicted genes (green arrow indicates orientation for each feature), the location of phage elements (orange blocks generated in Phaster), and GC skew from the average 61.45% GC (inner-most ring). The four outer rings show similarity to the *Ch_*R13 genome (blastn hits, *e*-value cuttoff = 0.1, Proksee): *C. haemolyticum* DSM 19808 (green), *C. haemolyticum* NRRL B11053 (brown), *C*. sp. Panama (blue), and *Chromobacterium subtsugae* PRAA4-1 (purple). (**B**) Maximum-likelihood phylogenetic tree (IQTree) for the *C. haemolyticum* group visualized in iTOL (red circles indicate 100 bootstrap support over 95 is shown) using 203 single-copy orthologous genes in GoToTree with the default settings (scale bar = substitutions per site). Location of the distantly related species, *C. subtsugae,* is shown, but the full phylogeny for the genus *Chromobacterium* is shown in Fig. S2. (**C**) Major genomic features of *Ch_*R13 compared with *C*. sp. Panama (Panama), DSM 19808, NRRL B-11053, and *C. subtsugae* PRAA4-1.

We selected an isolate designated Rain0013 for whole-genome sequencing using the PacBio platform. Results generated 371,442 reads with a mean length of 15,753 bp and a maximum length of 58,865 bp after correction. Assembly (455× coverage) yielded a single circular chromosome of 4,995,443 bp with a guanine-cytosine (GC) content of 61.45% and 4,538 predicted coding sequences (CDSs) (Fig. 1A). GoToTree recovered 203/203 single-copy orthologous genes from which we removed three that were duplicated (42). Phylogenetic analysis using these data and other publicly available genomes generated a tree that divided the genus *Chromobacterium* into two clades: one containing Rain0013 that we named the *C. haemolyticum* group and another containing all other sequenced isolates (Fig. S2). These results together with an average nucleotide identity (ANI) analysis at a cutoff of 95% (43) placed four described (*Chromobacterium aquaticum*, *Chromobacterium alkanivorans*, *Chromobacterium rhizoryzae*, and *Chromobacterium haemolyticum*) and one potentially undescribed species (designated as *C. haemolyticum* 2) in the *C. haemolyticum* group (Fig. 1B; Fig. S3). Rain0013 was identified with strong support as a strain of *C. haemolyticum* which includes other isolates such as DSM 19808 and NRRL B-11053 that have not previously been studied to assess whether they are also mosquitocidal. In contrast, *Chromobacterium* sp. Panama that is mosquitocidal (38) was identified as a strain of *C. rhizoryzae* along with *Chromobacterium* sp. Beijing, which was also isolated from a mosquito and was reported to inactivate dengue virus but was not reported to be mosquitocidal (44) (Fig. 1B; Fig. S3). Overall, genome size, number of CDSs, and metabolic pathways were similar among *Ch_*R13, DSM 19808, NRRL B-11053, and *Chromobacterium* sp. Panama (Fig. 1A and C).

### *Ch_*R13 and other members of the *C. haemolyticum* group share additional genomic features

We renamed Rain0013 *C. haemolyticum* Rain0013 (*Ch_*R13) and expanded our comparative analysis of the *C. haemolyticum* group by assessing synteny. Genome organization was broadly similar among species and strains with the exception of genomic islands (Fig. S4A). Directly comparing *Ch_*R13 to *Chromobacterium* sp. Beijing yielded the same trends, whereas dissimilarities in genome synteny were larger when compared with *Chromobacterium* sp. Panama due likely to poor genome assembly quality for the latter (61 contigs) (Fig. S4B). Shared synteny suggested central metabolic pathways are likely conserved in the *C. haemolyticum* group, whereas traits associated with genomic islands that can include pathogenicity factors are potentially more variable. However, querying the Virulence Factor Database (VFDB) indicated known virulence factors were largely conserved among *C. haemolyticum* group members and *C. subtsugae* which served as a more distantly related species in the genus (Table S1). Among these factors is the predicted synthesis of cyanide that *Chromobacterium* and select other genera produce via an *hcnABC* operon encoding a cyanide synthase (45). The only isolate lacking an *hcnABC* operon was strain C-61 (Table S1), but this could be an error due to its extremely fragmented genome (1118 contigs). All previously characterized *Chromobacterium* spp. encode Type 3 (T3SS) and Type 6 secretion systems (T6SS) (46–49) with this study indicating *Ch_*R13 encodes three T6SS operons while *Chromobacterium* sp. Panama, *Chromobacterium* sp. Beijing, and *C. haemolyticum* NRRL B-11053 encode four (Fig. S5). Taken together, *Ch_*R13 and all other members of the *C. haemolyticum* group likely produce a broad-spectrum toxin (cyanide) that binds cytochrome oxidase C in the electron transport chain of aerobic organisms (45) plus other virulence factors that could be introduced into multicellular animals or microbes through T3SS and T6SS.

### Larvae in AX cultures are more susceptible to *Ch_*R13 than larvae in CN cultures

**Fig 2:**
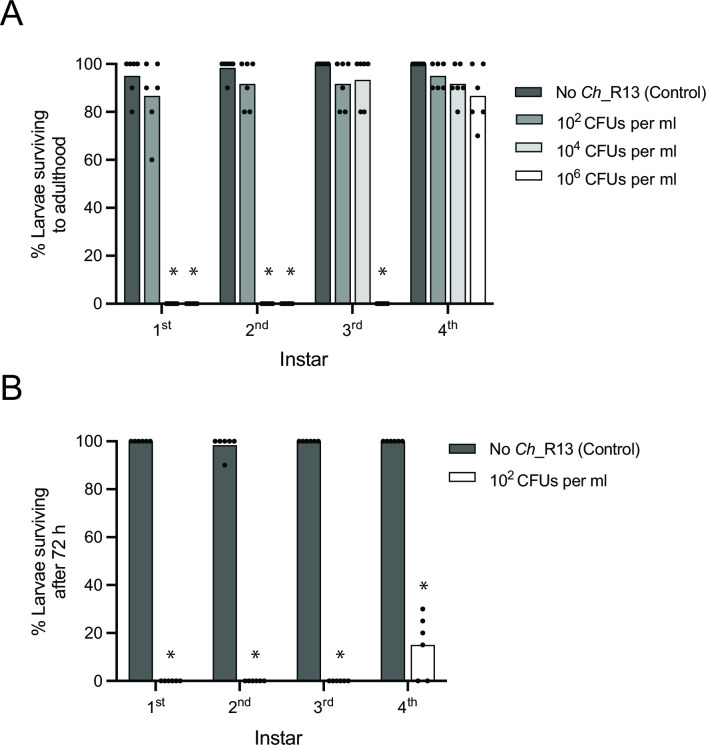
*Ch_*R13 causes lower mortality in CN than AX cultures of *A. aegypti* larvae. (**A**) Percentage of first–fourth instar *A. aegypti* in CN cultures that develop into adults when no *Ch*_R13 is added to culture wells (Control) versus when *Ch_*R13 is added at a starting density of 1 × 10^2^, 10^4^, or 10^6^ CFUs per mL. Each replicate is a culture well with 10 larvae (six replicates per treatment). Columns show mean values for each treatment with each replicate shown as solid circles. For each instar, an asterisk (*) indicates a significant difference for a given treatment when compared with the Control as determined by a Fisher’s exact test (*P* < 0.0001). (**B**) Percentage of first–fourth instar *A. aegypti* in AX cultures that are alive after 72 h when no *Ch*_R13 are added to culture wells (Control) versus when *Ch_*R13 is added at a starting of 10^2^ CFUs per mL. Columns, solid dots, and asterisks are defined as in (**A**).

We next assessed the mosquitocidal activity of *Ch_*R13 and related strains (Table S2) from the perspective of whether disease severity is affected by the community of microbes in container habitats. For these studies, we focused on *A. aegypti* for two reasons. First, we had earlier characterized by amplicon sequencing the container microbiota in our CN laboratory *A. aegypti* culture which predominantly consists of bacteria in the same phyla (Firmicutes, Actinobacteria, Betaproteobacteria, Gammaproteobacteria, and Bacteroidetes) as the microbial communities present in field containers (19, 22). Second, we had previously isolated many of these community members, while also establishing methods for producing AX cultures with no microbiota or GN cultures with a defined container microbiota (19, 29, 32). The community of bacteria in CN cultures increases to ~10^8^ CFUs per mL within 24 h of adding *A. aegypti* larvae to sterile water plus sterilized rearing diet which serves as the detritus source in laboratory cultures. The community of bacteria then persist at a density of ~10^8^ CFUs per mL until larvae pupate (30). We therefore established CN cultures where the resident microbiota was ~10^8^ CFUs per mL before adding *Ch_*R13 at three starting densities to containers where larvae were first–fourth instars. Most individuals at each instar developed into adults at the lowest starting density tested (1 × 10^2^ CFUs per mL) (Fig. 2A). Most first and second instars died, while most third and fourth instars developed into adults when *Ch_*R13 was added at an intermediate density (1 × 10^4^ CFUs per mL) (Fig. 2A). Only some fourth instars survived when *Ch_*R13 was added at the highest density (1 × 10^6^ CFUs per mL) (Fig. 2A). Thus, larvicidal activity increased with *Ch_*R13 starting density and early instars exhibited higher mortality than later instars. We then conducted the same assays in AX cultures with no container microbiota, which showed that all instars died within 2 days when *Ch_*R13 was added at 1 × 10^2^ CFUs per mL (Fig. 2B). Since *Ch*_R13 shared the same virulence factors with other strains of *C. haemolyticum*, we also tested DSM 19808 and NRRL B-11053 for larvicidal activity (Table S2). Both caused dose-dependent mortality in CN cultures containing *A. aegypti* first instars, whereas the lowest starting density tested (1 × 10^2^ CFUs per mL) killed all larvae in AX cultures (Fig. S6).

Given the preceding results, we compared growth of *Ch_*R13 in CN and AX cultures when introduced at a starting density of 1 × 10^2^ CFUs per mL. Colony counts showed that *Ch_*R13 increased in AX cultures to 10^8^ CFUs per mL in 30 h while a qPCR assay that measured the copy number of the single-copy *Ch_*R13 *gyrase* A gene (*gyr*A) generated similar abundance estimates at 8 and 20 h post-inoculation (Fig. S7A and B). We thus used this qPCR assay to estimate *Ch_*R13 in CN cultures, which showed that densities rose from 10^2^ to 10^4^ genome copies per mL by 8 h post-inoculation but thereafter ceased to further increase as observed in AX cultures (Fig. S7C). In contrast, colony counts detected no differences in the density of culturable bacteria at 8 and 20 h post-inoculation between CN cultures with no *Ch_*R13 and CN cultures to which 1 × 10^2^ CFUs per mL of *Ch_*R13 was added (Fig. S7D). Thus, *Ch_*R13 increased from a low starting density to much higher densities in AX than CN cultures which correlated with larval mortality also being higher in AX than CN cultures. However, inoculating CN cultures with a starting density of 1 × 10^6^ CFUs per mL overcame this constraint as evidenced by densities increasing to more than 10^8^ genome copies per mL by 92 h (Fig. S7E), which was consistent with a higher starting density of *Ch_*R13 also killing all CN first instars (see Fig. 2).

### Larvae in GN cultures are also less susceptible to *Ch_*R13

**Fig 3:**
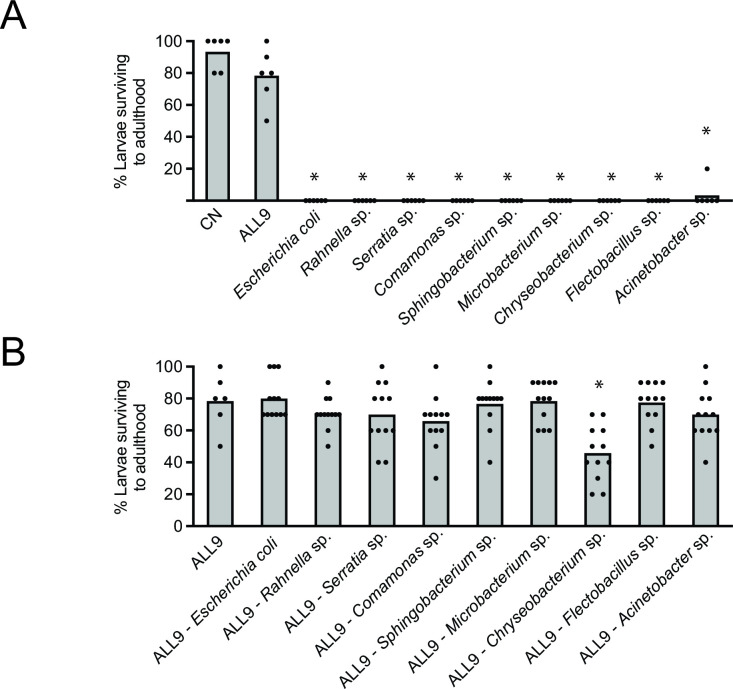
*Ch_*R13 causes lower mortality in GN cultures with a simplified nine-member community of commensal bacteria. (**A**) Percentage of first instar *A. aegypti* that develop into adults in CN cultures (Control), GN cultures with the ALL9 community, or GN cultures with only one member of the ALL9 community when *Ch_*R13 is added at a starting density of 1 × 10^2^ CFUs per mL. Columns show mean values for each treatment with values for each replicate shown as solid circles. An asterisk (*) indicates a significant difference for a given treatment when compared with the Control as determined by a Fisher’s exact test (*P* < 0.0001). (**B**) Percentage of first instar *A. aegypti* that develop into adults in GN cultures with the ALL9 community (Control) versus GN cultures lacking one member of the ALL9 community when *Ch_*R13 is added at a starting density of 1 × 10^2^ CFUs per mL. Columns, solid circles, and asterisks as defined in (**A**).

To assess whether diversity of the environmental microbiota affected *Ch_*R13 growth and disease severity, we took advantage of prior results where we created a simplified seven-member container microbiota (ALL7) consisting of seven taxonomically diverse species that we isolated from CN cultures (30). The ALL7 community grows to a stable density of ~10^8^ CFUs per mL in 24–36 h when added to an AX culture containing sterile water, sterilized rearing diet, and newly hatched *A. aegypti* larvae (30). Larval development time and size of resulting adults from cultures containing the ALL7 community do not differ from CN cultures indicating this simplified GN community comparably supports mosquito growth (30). In this study, we added *Comamonas* sp. and *Chryseobacterium* sp. that were also earlier isolated from our CN *A. aegypti* culture (19) to the ALL7 community, which created an ALL9 community that we added to AX cultures containing first instars (Table S2). We then added 1 × 10^2^ CFUs per mL of *Ch_*R13 24 h later. Most larvae in these GN cultures developed into adults, whereas most larvae died in 1–2 days if AX cultures were inoculated with only one member of the ALL9 community before introducing *Ch_*R13 (Fig. 3A). No reduction in survival occurred when AX cultures were inoculated with 8-member communities except in the case of omitting *Chryseobacterium* sp. which did reduce the percentage of larvae that survived to adulthood (Fig. 3B). Thus, the ALL9 and most eight-member communities had a similar protective effect as the more diverse microbiota in CN containers, but reducing diversity to a single member of the ALL9 community had no protective effect.

**Fig 4:**
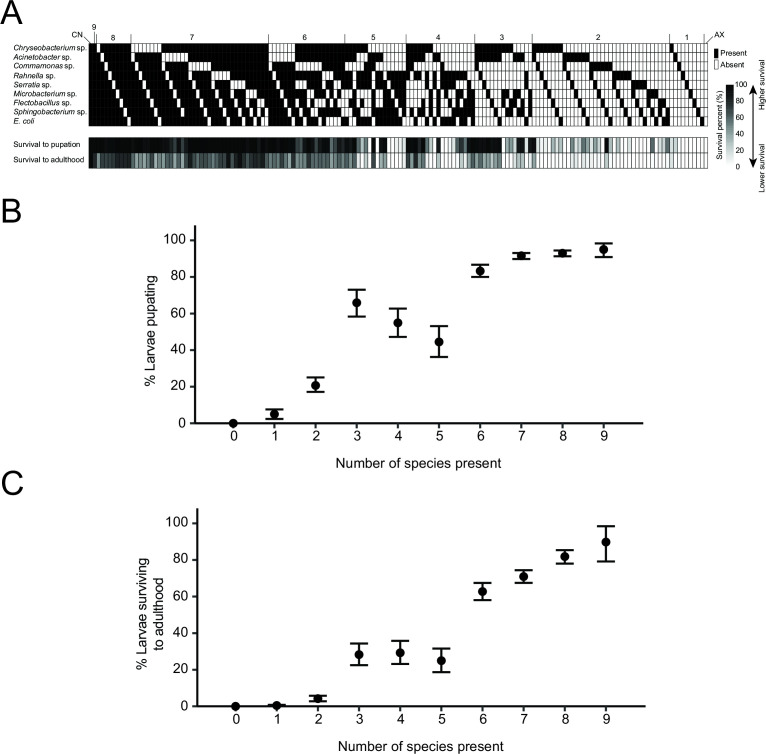
Larval mortality declines as diversity of the environmental bacterial community in the mosquito container habitat increases. (**A**) Schematic showing the percentage of *A. aegypti* larvae that survive to pupation or adulthood in AX cultures containing no environmental microbiota, GN cultures containing from one to eight species of the ALL9 community, the ALL9 community, or a CN community. For each treatment, the upper panel uses a black box to indicate a given species is present while a white box indicates a given species is absent. The lower panel shows percent survival to pupation or adulthood using a grayscale gradient from high (black) to low (white). A minimum of six biological replicates was conducted for each environmental community that was tested with the unit of replication being a culture well containing the environmental community of bacteria and a starting density of 10 larvae (first instars) to which 1 × 102 CFUs per mL *Ch_*R13 was added. (**B**) Graphs showing the percentages of larvae surviving to pupation (upper) or adulthood (lower) with 95% bootstrap confidence intervals when all treatments and replicates for a given environmental community size are combined. The percentage of larvae pupating or emerging as adults both significantly increase with diversity of the environmental microbiota (*P* < 0.0001; Kruskal-Wallis tests). Larvae in cultures with similar levels of commensal community diversity (i.e., low (zero, one, or two species), medium (three, four, or five species), or high (six, seven, eight, or nine species) were also grouped together and analyzed by pairwise Wilcoxon rank sum tests for multiple comparisons with Bonferroni correction (α ≤ 0.05). Different lowercase letters indicate the percentage of larvae pupating or emerging as adults significantly differed for the low-, medium-, and high-diversity communities.

We thus tested all combinations of two to seven member communities to ask if larval survival to pupation and adulthood in response to a low starting inoculum of *Ch_*R13 increased with diversity of the environmental community. Figure 4A summarizes the percentage of larvae that pupated and emerged as adults from AX cultures and GN cultures containing one to all members of the ALL9 community after adding 1 × 10^2^ CFUs per mL of *Ch_*R13. We also present these data by showing the percentage of larvae that survived to pupation or adulthood with 95% bootstrap confidence intervals when all treatments for a given environmental community size are combined (Fig. 4B). Results as presented in Fig. 4A qualitatively suggested the percentage of larvae that pupated or emerged as adults increases with diversity of the environmental microbiota, which was fully supported by analysis of the data as presented in Fig. 4B, which showed that pupation and adult emergence increased from the lowest diversity communities to the intermediate and highest diversity communities. We also conducted multivariate logistic regression analyses of the pupation and adult emergence data using two models: one that examined how individual species in our environmental microbiotas affected survival to the pupal and adult stages and another that examined all possible two-species interactions. We could not assess higher order interactions (i.e. three to nine species) because our data set, while large, was not large enough to do so. Individual species effects indicated each member of the ALL9 community increased the percentage of larvae that pupated and emerged as adults relative to no environmental microbiota being present, which was fully consistent with no larvae surviving to pupate or emerge as adults in the absence of an environmental microbiota (Tables S3 and S4). Several species interactions also significantly affected pupation and adult emergence with magnitudes that were equivalent or greater than the effects of individual species (Tables S3 and S4).

We noted from our multivariate logistic regression results that individual or two-species interactions among *Chryseobacterium* sp., *Comamonas* sp., and *Acinetobacter* sp. more strongly affected pupation and adult survival than most other members of the ALL9 community (Tables S3 and S4). We thus additionally compared a subset of the results in Fig. 4A that included seven member communities lacking *Escherichia coli*, *Rahnella* sp., *Serratia* sp., *Sphingobacterium* sp., *Flectobacillus* sp., and *Microbacterium* sp., which showed that survival to adulthood did not differ from the ALL9 community (Fig. S8). In contrast, survival was lower when seven-member communities lacked *Chryseobacterium* sp. or *Acinetobacter* sp., while no larvae survived in seven-member communities lacking *Chryseobacterium* sp. and *Acinetobacter* sp. or a six-member community lacking *Chryseobacterium* sp., *Acinetobacter* sp., and *Comamonas* sp. (Fig. S8). We also noted that a two-member community consisting of *Chryseobacterium* sp. and *Acinetobacter* sp. or a three-member community consisting of these species and *Comamonas* sp. also resulted in 35% and 60% survival, respectively, which was lower than that for the ALL9 community but higher than that for a three-member community like *Sphingobacterium* sp., *Microbacterium* sp., and *Flectobacillus* sp. where all larvae died (Fig. S8). As earlier found for CN cultures, we also determined that *Ch_*R13 grew to a lower density when inoculated into a GN culture containing the ALL9 community but grew to densities that were similar to an AX culture when inoculated into a GN culture containing *Sphingobacterium* sp., *Microbacterium* sp., and *Flectobacillus* sp. (Fig. S9A and B). Altogether, these results indicated the percentage of larvae that pupated and emerged as adults increased with diversity of the environmental microbiota, which was associated with *Ch_*R13 not growing to a density of 10^8^ CFUs per mL. The preceding experiments also suggested certain members of the ALL9 community (*Acinetobacter* sp., *Chryseobacterium* sp., and *Comamonas* sp.) more strongly promoted pupation and survival to the adult stage than others.

### *Ch_*R13 produces sufficient hydrogen cyanide at high densities to kill *A. aegypti* larvae

**Fig 5:**
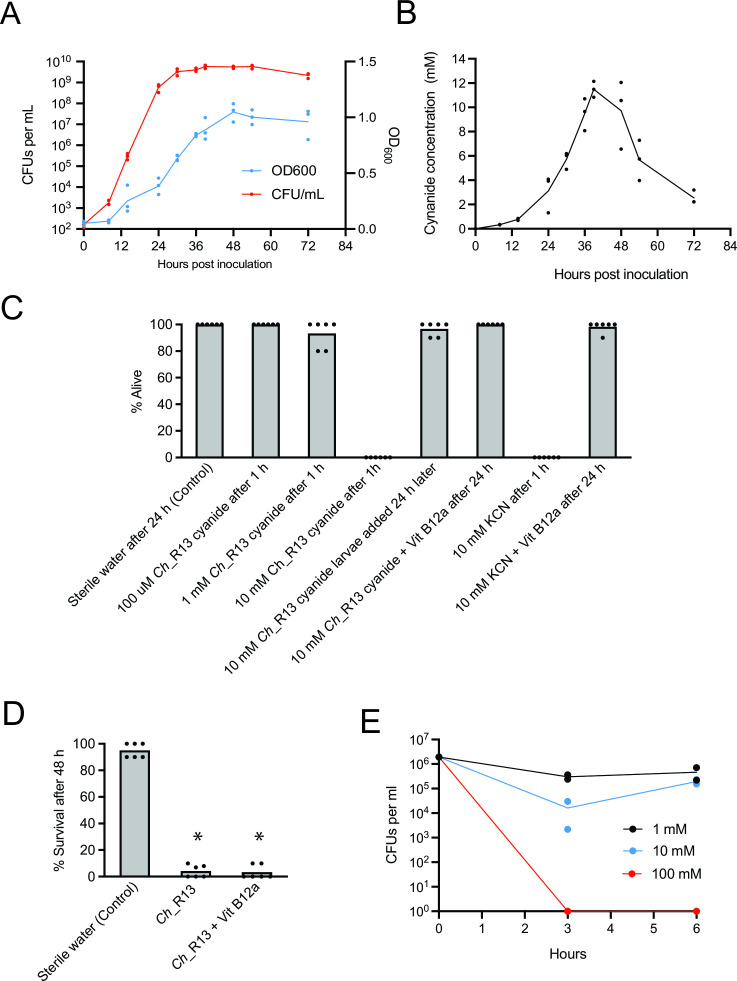
*Ch_*R13 at densities above 10^8^ CFUs per mL produce cyanide concentrations that kill *A. aegypti* first instars. (**A**) Growth curves showing OD600 and CFUs per mL of *Ch_*R13 in AX cultures that mimicked rearing conditions used for larvae. Growth was assessed in three independent assays with solid circles indicating values for each replicate. (**B**) Cyanide concentrations in the water of the cultures shown in (**A**). (**C**) Percentage of first instar *A. aegypti* that were alive 1 h or 24 h after being put in sterile water (Control), filter-sterilized water from a *Ch_*R13 culture, or a KCN stock solution with different starting concentrations of cyanide plus or minus 10 mM hydroxocobalamin (vitamin B12a). Columns and solid circles are defined as in Fig. 2. An asterisk (*) indicates a significant difference for a given treatment when compared with the Control as determined by a Fisher’s exact test (*P* < 0.0001). (**D**) Percentage of first instar *A. aegypti* that were alive 48 h after being put in sterile water (Control), sterile water with *Ch_*R13 at a starting density of 10^2^ CFUs per mL, or sterile water with *Ch_*R13 at a starting density of 10^2^ CFUs per mL plus 10 mM vitamin B12a. Columns, solid circles, and asterisks as defined in (**C**). (**E**) CFUs per mL of bacteria that grow on LB plates in open CN cultures 0–6 h after adding 1–100 mM cyanide.

Given the correlation between *Ch_*R13 density and mortality of *A. aegypti* larvae, we noted recent findings showing that *Chromobacterium* sp. Panama produces and releases sufficient cyanide at high densities to kill *Anopheles gambiae* mosquito larvae (39). Culturing *Ch_*R13 under conditions used to rear *A. aegypti* larvae showed that cyanide concentrations in the water increased to 10 mM before declining, which was several hours after bacteria reached a stationary density of 10^9^ CFUs per mL (Fig. 5A and B). In contrast, no cyanide was detected in water from three independently established CN cultures 36 h post-inoculation where bacteria culturable on LB plates also reach a density of 10^9^ CFUs per mL indicating the commensal microbes present in CN cultures do not produce measurable amounts of cyanide. We next measured the concentration of cyanide that kills *A. aegypti* first instars by serially diluting filter-sterilized water containing 10 mM cyanide from a *Ch_*R13 culture or a potassium cyanide (KCN) stock solution followed by adding 1-mL volumes to open 24-well culture plates. All first instars added to wells containing 10 mM cyanide died within 1 h, whereas concentrations ≤ 1 mM resulted in few or no larvae dying over the same period (Fig. 5C). In contrast, adding filter-sterilized water from a *Ch_*R13 culture with a starting cyanide concentration of 10 mM to open wells but waiting 24 h before introducing larvae resulted in almost no mortality (Fig. 5C). We determined that cyanide concentrations in these wells had fallen below 1 mM, which was consistent with the presence of primarily hydrogen cyanide which is volatile (50). Adding the cyanide scavenger hydroxocobalamin (vitamin B12a) to filter-sterilized water containing 10 mM cyanide also nearly fully inhibited larval mortality but adding hydroxocobalamin to AX cultures inoculated with 10^2^ per mL *Ch_*R13 did not (Fig. 5C and D). Lastly, we observed that cyanide in water dose-dependently reduced the density of culturable bacteria from CN cultures after a 3-h incubation period, which remained suppressed at 6 h at a starting concentration of 100 mM but recovered to starting densities at a starting concentration of 10 mM (Fig. 5E). Collectively, these assays suggested cyanide was the primary if not only factor in filter-sterilized water from high-density *Ch_*R13 cultures that killed *A. aegypti* larvae given near complete inhibition by hydroxocobalamin. Cyanide from a high-density *Ch_*R13 culture could also reduce the density of commensal microbes present in CN cultures. However, living *Ch_*R13 potentially produce other virulence factors that contribute to larval mortality given hydroxocobalamin did not inhibit larval mortality when *Ch_*R13 was present.

### *Ch_*R13 systemically infects larvae through the midgut

**Fig 6:**
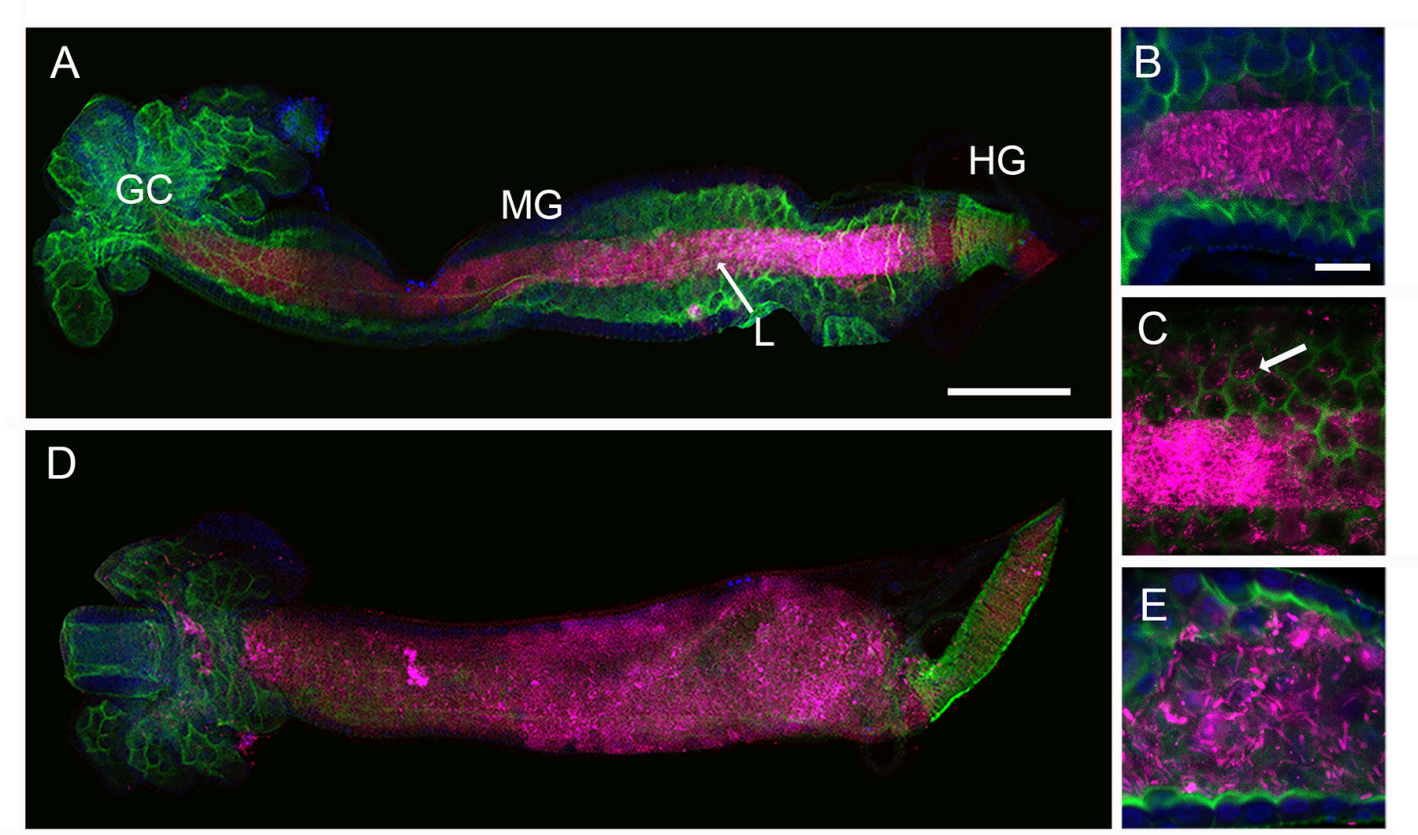
*Ch_*R13^E2-SpR^ systemically infects *A. aegypti* larvae. AX cultures containing first instars were inoculated with 1 × 10^2^ CFUs per mL followed by dissection of larvae that were fixed and stained with phalloidin to visualize the borders of gut cells and DAPI to visualize gut cell nuclei. (**A**) Low-magnification confocal image of the digestive tract 8 h post-inoculation. *Ch_*R13^E2-SpR^ (red) is restricted to the gut lumen (L). Gastric caeca (GC), midgut (MG), and hindgut (HG). Scale bar = 100 μm. (**B**) Higher magnification image of the midgut from a larva 8 h post-inoculation showing all *Ch_*R13^E2-SpR^ in the lumen. Scale bar = 20 μm. (**C**) Higher magnification image of the midgut from a larva 18 h post-inoculation showing *Ch_*R13^E2-SpR^ has breached the peritrophic matrix lining the midgut lumen and is now present in midgut cells (arrow). (**D**) Low-magnification image of the digestive tract 24 h post-inoculation. *Ch_*R13^E2-SpR^ is present throughout the midgut. (**E**) Higher magnification image of the midgut from a larva 24 h post-inoculation showing an abundance of *Ch_*R13^E2-SpR^ with loss of phalloidin staining indicating loss of structural integrity of midgut cells.

To further examine mortality associated with the presence of *Ch_*R13, we produced *Ch_*R13^E2-SpR^ that was visualized through expression of E2-Crimson. Inoculating AX and CN cultures containing first instars with 1 × 10^2^ or 1 × 10^6^ CFUs per mL of *Ch_*R13^E2-SpR^ resulted in the same outcomes as previous assays using *Ch_*R13 with all AX larvae dying in 1–2 days, while CN larvae usually survived at a low starting inoculum but died at a high starting inoculum (Fig. S10A). As earlier noted, these patterns further correlated with *Ch_*R13^E2-SpR^ growing to high densities in AX cultures but requiring a high starting inoculum to grow to high densities in CN cultures. As earlier noted, commensal bacteria in CN or GN cultures circulate between the larval gut and water (27, 28). In the midgut (MG), commensal bacteria in CN cultures are further restricted to the lumen, which is bounded by a peritrophic matrix consisting of glycoproteins and chitin (28). When *Ch_*R13^E2-SpR^ was added to AX cultures at 10^2^ CFUs per mL, we likewise observed that it was restricted to the midgut lumen at 8 h post-inoculation but by 18 h had breached the peritrophic matrix and were in contact with midgut cells (Fig. 6A through C). At 24 h, *Ch_*R13^E2-SpR^ was observed throughout the midgut with evidence of midgut cell lysis (Fig. 6D and E). *Ch_*R13^E2-SpR^ was thereafter also observed in the hemocoel of larvae which was followed by death. The same assay conducted in the presence of hydroxocobalamin indicated *Ch_*R13^E2-SpR^ also breached the midgut by 18 h and systemically infected larvae. In contrast, *Ch_*R13^E2-SpR^ remained in the gut lumen over the same period when CN cultures were inoculated with 10^2^ CFUs per mL. Vital dye staining using propidium iodide (PI) detected no loss of midgut cell viability at 8 h post-inoculation in AX or CN larvae, whereas most midgut cells in AX but not CN larvae were PI stained at 24 h (Fig. S10B). Qualitative assays additionally indicated *Ch_*R13, *C. haemolyticum* DSM 19808, and *C. haemolyticum* NRRL B-11053 exhibited both general protease and chitinase activity consistent with the ability to breach the peritrophic matrix and enter midgut cells (Fig. S11).

## DISCUSSION

Identifying a container habitat with mosquitocidal activity arose from monitoring breeding sites for the presence of *Aedes* and *Culex* larvae rather than deliberately searching for pathogens. Nonetheless, identifying *Ch_*R13 was of interest from two perspectives. First, four other *Chromobacterium* have been identified (*Chromobacterium* sp. Panama, *Chromobacterium violaceum*, *Chromobacterium vaccinii*, and *C. aquaticum*) that also exhibit mosquitocidal activity (38–40). However, two of these species (*C. violaceum* and *C. haemolyticum*) and several other *Chromobacterium* are also reported to infect and cause disease in other animals including humans while also exhibiting a range of anti-microbial activities (46, 49, 51–57). Yet, other studies report that *C. aquaticum* functions as a beneficial probiotic in fish (58) while *Chromobacterium* sp. Beijing exhibits activity that potentially confers resistance to an arbovirus in adult mosquitoes (44). Collectively, these findings suggest either *Chromobacterium* species or strains have variable host ranges as pathogens or can broadly cause disease but unknown factors determine infection risks and disease severity or potentially enable some *Chromobacterium* to benefit animal hosts. We thus expanded our analysis of *Ch_*R13 to include a phylogenetic analysis of the genus and a comparison of virulence gene inventories among isolates that are known to be mosquitocidal versus isolates that are unknown to kill mosquitoes. Our second reason for conducting this study derived from observing that the mosquitocidal activity of *Ch_*R13 was not present in neighboring containers we surveyed for mosquito larvae, suggesting conditions existed that were unfavorable to *Ch_*R13 invasion, persistence, or causing fatal disease. Increased diversity in the host-specific gut microbiotas of animals and rhizospheres of plants confers resistance to some pathogens (59–61). Whether environmental microbial communities also potentially affect animal pathogens is largely unknown (62), but we reasoned they could due to water readily transporting microbes and general toxins. We thus took advantage of established methods for producing AX and GN mosquito cultures to assess how diversity of the aquatic microbiota affects disease severity in *A. aegypti* caused by *Ch_*13.

Our phylogenetic and ANI analyses identify *Ch_*R13 as a strain of *C. haemolyticum* along with NRRL B-11053 and DSM 19808 that we show are also mosquitocidal. We further identify *Chromobacterium* sp. Panama as a strain of *C. rhizoryzae* which together with *C. haemolyticum*, *C. aquaticum*, and *C. alkanivorans* comprise an assemblage we refer to as the *C. haemolyticum* group. Most if not all members of the *C. haemolyticum* group share a common inventory of virulence genes including an *hcnABC* operon that produces cyanide. Functional assays reported in this study for *Ch_*R13 combined with earlier results (39) thus suggest all members of the *C. haemolyticum* group are potentially mosquitocidal. This conclusion also likely extends to the genus as a whole given our phylogenetic analysis and evidence that *C. violaceum*, *C. vaccinii*, *Chromobacterium piscinae*, and *C. subtsugae* produce cyanide plus other virulence factors that kill mosquitoes or other insects (37, 40, 52, 63–65). Many of these species also cause disease in other organisms including other types of insects, suggesting most *Chromobacterium* have the potential to be broadly pathogenic by secreting a general toxin (cyanide) and producing other cell-targeted virulence factors delivered by T3SS or T6SS. The broad virulence properties of *Chromobacterium* spp. potentially limit their use for controlling mosquito populations but the virulence factors produced may have broader applications.

Why *Chromobacterium* spp. produce a broad inventory of virulence factors is incompletely understood but is potentially linked to inhabiting soil or aquatic habitats where saprophytes in polymicrobial communities compete for resources (46, 49). Some *Chromobacterium* have further been shown to use promiscuous, cross-species quorum sensing (QS) to control the production of virulence factors that kill competitors (65). A simplified dual-species assay shows QS controls the density-dependent production of cyanide and antibiotics by *C. subtsugae* with the former being the primary factor that enables it to outcompete other saprophytes (66, 67). In turn, QS-deficient mutants of *C. violaceum* produce less cyanide (68), while cyanide produced by other *Chromobacterium* spp. reduce the growth of other aerobic microbes (45, 55, 69). However, *C. violaceum* also produces other antimicrobial factors whose synthesis and secretion are linked to QS, while the broad-spectrum antibacterial activity of *C. haemolyticum* W15 requires direct contact with competing bacteria suggesting roles for T3SS and/or T6SS in delivery (49, 70). Thus, resource competition with other microbes potentially contributes to regulating cyanide production that kills animals like mosquito larvae which also provide resources for growth.

Assessing the role of intra- or cross-species QS in cyanide production was not the objective of this study, but our results nonetheless indicate *Ch_*R13 must reach a threshold density of ~10^8^ CFUs per mL to produce cyanide concentrations that kill *A. aegypti* larvae. This threshold was attained in AX cultures inoculated with a low starting density or CN cultures inoculated with a high starting density of *Ch_*R13. In contrast, CN or GN cultures inoculated with a low starting density result in *Ch_*R13 remaining below 10^5^ CFUs per mL which produced cyanide concentrations that were below fatal levels. That hydroxocobalamin inhibits mortality of *A. aegypti* larvae indicates cyanide is the primary larvicidal factor in filter-sterilized water from high-density *Ch_*R13 cultures. In contrast, hydroxocobalamin does not inhibit mortality when AX cultures are inoculated with a low starting inoculum of *Ch_*R13 suggesting a role for other, currently unknown, virulence factors in mosquito mortality. This conclusion is also supported by observing that *Ch_*R13^E2-SpR^ infects larvae through the midgut with general protease and chitinase activity potentially facilitating entry through the peritrophic matrix.

Our results indicate diversity of the aquatic microbiota additionally influences whether a low starting inoculum of *Ch_*R13 reaches a threshold density that kills *A. aegypti* larvae. Assays using the ALL9 community indicate communities of ~9 commensal bacteria reduce larval mortality, but further reducing community diversity progressively enables *Ch_*R13 to increase in density to levels that approach densities in AX cultures that are fatal to larvae across all instars. However, our results also suggest certain members of the ALL9 community (i.e., *Chryseobacterium* sp. and *Acinetobacter* sp.) are more important than others in limiting *Ch_*R13 densities to below fatal levels for *A. aegypti*. Outcomes are potentially explained by a decrease in the competitive ability of *Ch_*R13 in more diverse communities due to reduced rates of resource consumption (60, 61) or direct antagonism by community members like *Chryseobacterium* sp. and *Acinetobacter* sp. Laboratory models developed to mimic interactions in polymicrobial communities indicate both environmental conditions and community composition can positively or negatively modulate QS activation of anti-microbial molecules including cyanide production (71, 72). We thus speculate that in our field sites, *Ch_*R13 grew to higher densities in the “Rain” container due to a higher starting inoculum, the composition of the microbial community, and/or other culture conditions that favored *Ch_*R13 growth over other microbes. That early instar mosquito larvae are more susceptible to fatal infection than late instar larvae additionally suggests a role for larval stage in susceptibility to fatal infection by *Ch_*R13, which is consistent with a number of studies in the insect literature reporting that early instars are often more susceptible to bacterial and viral pathogens than older instars (73–76). The underlying mechanisms for these differences are incompletely understood but potentially involve the nutritional state and physiological changes in the midgut (77, 78).

In summary, our results suggest most *Chromobacterium* have the potential to cause disease in a range of organisms but diversity of environmental microbial communities influences disease risk to mosquito larvae by modulating pathogen growth. In turn, these findings potentially extend to other aquatic animals including different types of beneficial and pest insects.

## MATERIALS AND METHODS

### Analysis of *Ch_*R13 and other species used in the study

Water from an outdoor container in Athens, GA, USA, named “Rain,” was collected in September 2017 and returned to the laboratory where it was centrifuged, and the pelleted cells were cryopreserved as a glycerol stock at −80°C. Individual colony morphotypes were isolated and identified with 16S rRNA gene sequencing while the genome of isolate Rain0013 was sequenced using PacBio and assembled using Canu (79) and Circlator (80). Gene and metabolic pathways were annotated with the Prokaryotic Genomes Annotation Pipeline (81). Virulence factors were compared among publicly available *Chromobacterium* genomes using the Virulence Factor Database (82) and Island Compare (83). Genomic synteny was visualized with Proksee (84) and phylogenies assessed using GoToTree (42) and iTOL (85). Detailed descriptions of media and isolation conditions, extraction, sequence data, assembly parameters, and genomic analyses are available in the supplemental Materials and Methods. Other species used in the study are summarized in Table S2.

### Larvicidal assays

Addition of laboratory-reared mosquito larvae to “Rain” container water resulted in rapid mortality. In controlled bioassays, bacteria were added at a starting density of 1 × 10^2^–1×10^6^ CFUs per mL to conventional, gnotobiotic, or axenic cultures containing *A. aegypti* larvae (first–fourth instars) followed by determining the number of alive versus dead larvae, which was apparent because living larvae are highly active, while dead larvae are immobile and rapidly discolor. AX larvae were produced as earlier described (19, 32). Bacteria comprising the ALL9 community were isolated from CN cultures (19, 29, 30) (Table S2). Other *C. haemolyticum* strains were obtained from stock centers (Agricultural Research Service Culture Collection (Northern Regional Research Laboratory (NRRL)) and Deutsche Sammlung von Mikroorganismen und Zellkulturen (DSMZ)) (Table S2). Defined communities of commensal microbes were produced by adding one or members of the ALL9 community to AX cultures (30). Specific information about mosquito rearing, bacterial cultures, and larvicidal assay conditions is available in the supplemental Materials and Methods.

### Virulence factor assays

Cyanide concentrations in *Ch_*R13 or CN cultures were measured by an established assay (86). Hydroxocobalamin was used to inactivate cyanide. Protease and chitinase activities were assayed with milk and colloidal chitin agar plates (87). pBTK570 encoding the E2-Crimson gene was transformed into *Ch_*R13, and the resulting fluorescent strain (*Ch_*R13^E2-SpR^) was used in visualizing infection in mosquitoes. Detailed procedures for transformation, microscopy, bioassays using cyanide, and enzymatic assays are available in the supplemental Materials and Methods.

### Data analysis

Data were organized in Excel files with graphs generated using GraphPad Prism (9.0) or R v4.1.0 (https://www.r-project.org/). Survival data were analyzed by Bonferroni-corrected pairwise Fisher’s exact tests that compared different treatments with a designated control. Bacterial abundance data were analyzed by *t*-test or one-way analysis of variance followed by a post hoc Dunnett’s test that compared treatments to a designated control. Survival of GN larvae with different commensal bacteria and *Ch_*R13 were compared by pooling data across treatments within a given diversity group and then interpreting groups with nonoverlapping 95% bootstrap confidence intervals based on 1,000 bootstrap replicates estimated using the *boot*() function in R. Follow-up comparisons of larval survival in cultures containing low (zero to two species), medium (three to five species), or high (six to nine species) levels of commensal community diversity were then made using a nonparametric Kruskal-Wallis test followed by pairwise Wilcoxon rank sum tests for multiple comparisons with Bonferroni correction. Contributions of individual ALL9 community members and those of interactions between different commensal species were assessed using multivariate logistic regression models estimated using the *glmer*() and *glm*() functions in R.

## Data Availability

The genome for *Chromobacterium haemolyticum* Rain 0013 (Ch_R13) is deposited in the National Center for Biotechnology Information (NCBI) GenBank under the BioProject PRJNA484200.

## SUPPLEMENTAL MATERIAL

Supplemental material mbio.02726-23-s0001.pdf Supplemental tables, figures, and methods.

ASM does not own the copyrights to Supplemental Material that may be linked to, or accessed through, an article. The authors have granted ASM a non-exclusive, world-wide license to publish the Supplemental Material files. Please contact the corresponding author directly for reuse.
