# Supplementary material for: Increased environmental microbial diversity reduces the disease risk of a mosquitocidal pathogen
Source: mBio. 2023 Dec 6;15(1):e02726-23. doi: 10.1128/mbio.02726-23 (PMC10790785; doi:10.1128/mbio.02726-23)
Supplement: Supplemental material — Supplemental tables, figures, and methods. [file mbio.02726-23-s0001.pdf]

SI Appendix (SI Tables, Figures, Methods)

Table S1. Virulence factors in the VFDB database identified in *Ch*_R13 and other strains of *C. haemolyticum*.

			C. subtsugae	*C. haemolyctium* group																							
VFclass	Virulence factors	Related genes	PRAA4-1	aquaticum DSM_19852	LK1	*alkanivorans* IITR-71	LK11	Panama	Beijing	*rhizoryzae* 32279	HSC-31F16	H4137	WI5	Rain0013	H5244	IR17	DSM_19808	Bb2	*rhizoryzae* JP2-74	NRRL_B-11053	IRSS-001	H3973	C-61	CH06-BL	T124	ZR-1	WJ-5
Adherence	Adhesion and penetration protein	app	-	-	-	-	-	-	-	-	-	-	-	-	-	-	-	-	-	-	-	-	-	-	-	-	-
	LOS sialylation	lst	-	-	-	-	-	-	-	-	-	-	-	-	-	-	-	-	-	-	-	-	-	-	-	-	-
	LOS synthesis	kdtA/waaA	MY55_11690	LQT98_RS09920	VI26_RS12670	JW897_RS13585	VK98_RS04190	DB032_22145	HQN78_RS18845	JC616_RS24180	HA052_RS08810	B0T39_RS13915	JOS77_RS01425	DXT74_03130	B0T45_RS17760	AWH52_RS09165	BR71_RS10220	IEZ30_RS23565	D1345_RS22765	DBB33_11955	MOV80_RS18145	B0T40_RS10745	CC61_RS21090	GYH29_RS23800	LF72_RS18495	JD974_RS15305	J1C50_RS17320
		lgtA	-	-	-	-	-	-	-	-	-	-	-	-	-	-	-	-	-	-	-	-	-	-	-	-	-
		lgtB	-	-	-	-	-	-	-	-	-	-	-	-	-	-	-	-	-	-	-	-	-	-	-	-	-
		lgtC	-	-	-	-	-	-	-	-	-	-	-	-	-	-	-	-	-	-	-	-	-	-	-	-	-
		lgtD	-	-	-	-	-	-	-	-	-	-	-	-	-	-	-	-	-	-	-	-	-	-	-	-	-
		lgtE	-	-	-	-	-	-	-	-	-	-	-	-	-	-	-	-	-	-	-	-	-	-	-	-	-
		lgtF	-	-	-	-	-	-	-	-	-	-	-	-	-	-	-	-	-	-	-	-	-	-	-	-	-
		lgtG	-	-	-	-	-	-	-	-	-	-	-	-	-	-	-	-	-	-	-	-	-	-	-	-	-
		lgtH	-	-	-	-	-	-	-	-	-	-	-	-	-	-	-	-	-	-	-	-	-	-	-	-	-
		rfaC	MY55_11685	-	-	-	-	-	-	-	-	-	-	-	-	-	-	-	-	-	-	-	-	-	-	-	-
		rfaF	MY55_14355	LQT98_RS04250	VI26_RS04605	JW897_RS18725	VK98_RS00590	DB032_06560	HQN78_RS08635	JC616_RS13520	HA052_RS14900	B0T39_RS01630	JOS77_RS24350	DXT74_12740	B0T45_RS17685	AWH52_RS20965	BR71_RS19980	IEZ30_RS08470	D1345_RS08185	DBB33_14090	MOV80_RS15470	B0T40_RS00250	CC61_RS22430	GYH29_RS08905	LF72_RS12665	JD974_RS03485	J1C50_RS03485
		rfaK	-	-	-	-	-	-	-	-	-	-	-	-	-	-	-	-	-	-	-	-	-	-	-	-	-
	Neisseria adhesion A	nadA	-	-	-	-	-	-	-	-	-	-	-	-	-	-	-	-	-	-	-	-	-	-	-	-	-
	Phosphoethanolamine modification	lptA	-	-	-	-	-	-	-	-	-	-	-	-	-	-	-	-	-	-	-	-	-	-	-	-	-
	Type IV pili	pilC	-	-	-	-	-	-	-	-	-	-	-	-	-	-	-	-	-	-	-	-	-	-	-	-	-
		pilD	-	LQT98_RS11500	VI26_RS09820	JW897_RS02835	VK98_RS11640	DB032_17440	-	JC616_RS19795	HA052_RS16985	B0T39_RS05845	JOS77_RS15860	DXT74_06715	B0T45_RS11450	AWH52_RS12310	BR71_RS12885	IEZ30_RS19760	D1345_RS19060	DBB33_20970	MOV80_RS07560	B0T40_RS01690	CC61_RS05455	GYH29_RS19845	LF72_RS09525	JD974_RS10770	J1C50_RS13085
		pilE	-	-	-	-	-	-	HQN78_RS15940	-	-	B0T39_RS10140	-	DXT74_00710	-	-	-	-	-	-	-	-	-	-	-	-	-
		pilF	MY55_09480	LQT98_RS11510	VI26_RS09810	JW897_RS02825	VK98_RS11630	DB032_17450	HQN78_RS23020	JC616_RS19805	HA052_RS16995	B0T39_RS05835	JOS77_RS15850	DXT74_06705	B0T45_RS11460	AWH52_RS12320	BR71_RS12895	IEZ30_RS19770	D1345_RS19070	DBB33_20980	MOV80_RS07570	B0T40_RS01680	CC61_RS05445	GYH29_RS19855	LF72_RS09535	JD974_RS10760	J1C50_RS13095
		pilG	MY55_09485	LQT98_RS11505	VI26_RS09815	JW897_RS02830	VK98_RS11635	DB032_17445	HQN78_RS23025	JC616_RS19800	HA052_RS16990	B0T39_RS05840	JOS77_RS15855	DXT74_06710	B0T45_RS11455	AWH52_RS12315	BR71_RS12890	IEZ30_RS19765	D1345_RS19065	DBB33_20975	MOV80_RS07565	B0T40_RS01685	CC61_RS05450	GYH29_RS19850	LF72_RS09530	JD974_RS10765	J1C50_RS13090
		pilH	-	-	-	-	-	-	-	-	-	-	-	-	-	-	-	-	-	-	-	-	-	-	-	-	-
		pilI	-	-	-	-	-	-	-	-	-	-	-	-	-	-	-	-	-	-	-	-	-	-	-	-	-
		pilJ	-	-	-	-	-	-	-	-	-	-	-	-	-	-	-	-	-	-	-	-	-	-	-	-	-
		pilK	-	-	-	-	-	-	-	-	-	-	-	-	-	-	-	-	-	-	-	-	-	-	-	-	-
		pilM	-	-	-	-	-	-	-	-	-	-	-	-	-	-	-	-	-	-	-	-	-	-	-	-	-
		pilN	-	-	-	-	-	-	-	-	-	-	-	-	-	-	-	-	-	-	-	-	-	-	-	-	-
		pilO	-	-	-	-	-	-	-	-	-	-	-	-	-	-	-	-	-	-	-	-	-	-	-	-	-
		pilP	-	-	-	-	-	-	-	-	-	-	-	-	-	-	-	-	-	-	-	-	-	-	-	-	-
		pilQ	MY55_06005	LQT98_RS06280	VI26_RS09155	JW897_RS01980	VK98_RS10840	DB032_18305	HQN78_RS21915	JC616_RS21050	HA052_RS12675	B0T39_RS18025	JOS77_RS15010	DXT74_05910	B0T45_RS03440	AWH52_RS12860	BR71_RS07570	IEZ30_RS20570	D1345_RS19815	DBB33_02045	MOV80_RS14195	B0T40_RS18385	CC61_RS18770	GYH29_RS20725	LF72_RS02965	JD974_RS13875	J1C50_RS06165
		pilS	-	-	-	-	-	-	-	-	-	-	-	-	-	-	-	-	-	-	-	-	-	-	-	-	-
		pilT2	MY55_03875; MY55_12510	LQT98_RS21825	VI26_RS18900	JW897_RS07130; JW897_RS14405	VK98_RS01670; VK98_RS03810	DB032_08165; DB032_21355	HQN78_RS11135; HQN78_RS19690	JC616_RS07210; JC616_RS23365	HA052_RS01410; HA052_RS09875	B0T39_RS14205; B0T39_RS18120	JOS77_RS13005; JOS77_RS17960	DXT74_03920; DXT74_18830	B0T45_RS14220; B0T45_RS17125	AWH52_RS05915; AWH52_RS11680	BR71_RS05930; BR71_RS15240	IEZ30_RS06040; IEZ30_RS22785	D1345_RS06065; D1345_RS21985	DBB33_05260; DBB33_11185	MOV80_RS00670; MOV80_RS04705	B0T40_RS14335; B0T40_RS20445	CC61_RS20545	GYH29_RS06670; GYH29_RS23015	LF72_RS00780; LF72_RS10945	JD974_RS01420; JD974_RS05570	J1C50_RS01420; J1C50_RS07955
		pilT	MY55_03880	LQT98_RS21820	VI26_RS18895	JW897_RS14400	VK98_RS03815	DB032_21360	HQN78_RS19685	JC616_RS23370	HA052_RS01415	B0T39_RS18125	JOS77_RS13000	DXT74_03915	B0T45_RS14225	AWH52_RS11675	BR71_RS15235	IEZ30_RS22790	D1345_RS21990	DBB33_11190	MOV80_RS04700	B0T40_RS14330	-	GYH29_RS23020	LF72_RS00775	JD974_RS01425	J1C50_RS01425
		pilU	-	-	-	-	-	-	-	-	-	-	-	-	-	-	-	-	-	-	-	-	-	-	-	-	-
		pilV	-	-	-	-	-	-	-	-	-	-	-	-	-	-	-	-	-	-	-	-	-	-	-	-	-
		pilW	-	-	-	-	-	-	-	-	-	-	-	-	-	-	-	-	-	-	-	-	-	-	-	-	-
		pilX	-	-	-	-	-	-	-	-	-	-	-	-	-	-	-	-	-	-	-	-	-	-	-	-	-
		pilZ	-	-	-	-	-	-	-	-	-	-	-	-	-	-	-	-	-	-	-	-	-	-	-	-	-
Efflux pump	FarAB	farA	MY55_06265	LQT98_RS05890	VI26_RS08895	JW897_RS01710	VK98_RS06130	DB032_18560	HQN78_RS21585	JC616_RS21310	HA052_RS12935	B0T39_RS18470	JOS77_RS14750	DXT74_05650	B0T45_RS03180	AWH52_RS13120	BR71_RS07830	IEZ30_RS20815	D1345_RS20070	DBB33_01805	MOV80_RS13950	B0T40_RS12325	CC61_RS06795	GYH29_RS20985	LF72_RS02705	JD974_RS14135	J1C50_RS05905
		farB	MY55_06270	LQT98_RS05885	VI26_RS08890	JW897_RS01705	VK98_RS06125	DB032_18565	HQN78_RS21580	JC616_RS21315	HA052_RS12940	B0T39_RS18475	JOS77_RS14745	DXT74_05645	B0T45_RS03175	AWH52_RS13125	BR71_RS07835	IEZ30_RS20820	D1345_RS20075	DBB33_01800	MOV80_RS13945	B0T40_RS12320	CC61_RS06790	GYH29_RS20990	LF72_RS02700	JD974_RS14140	J1C50_RS05900
	MtrCDE	mtrC	-	-	-	-	-	-	-	-	-	-	-	-	-	-	-	-	-	-	-	-	-	-	-	-	-
		mtrD	MY55_03335; MY55_04530; MY55_13875; MY55_21805	LQT98_RS00560; LQT98_RS01310; LQT98_RS10000; LQT98_RS11255; LQT98_RS14155; LQT98_RS20795; LQT98_RS20800	VI26_RS06715; VI26_RS12440; VI26_RS15810; VI26_RS15815; VI26_RS16580; VI26_RS21875	JW897_RS02105	VK98_RS10995	DB032_10115; DB032_11105; DB032_14875; DB032_18150; DB032_19190; DB032_20395	HQN78_RS03290; HQN78_RS08090; HQN78_RS09960; HQN78_RS20035; HQN78_RS20950; HQN78_RS22075	JC616_RS08630; JC616_RS10000; JC616_RS14845; JC616_RS20885; JC616_RS21940; JC616_RS23020	HA052_RS00045; HA052_RS01090; HA052_RS25760; HA052_RS26010; HA052_RS26320	B0T39_RS02070; B0T39_RS03455; B0T39_RS04495; B0T39_RS23175; B0T39_RS24405	JOS77_RS13360; JOS77_RS14250; JOS77_RS17115; JOS77_RS20405; JOS77_RS23905	DXT74_04280; DXT74_05140; DXT74_11955; DXT74_13185; DXT74_17815	B0T45_RS01830; B0T45_RS02680; B0T45_RS03590; B0T45_RS21180; B0T45_RS22810	AWH52_RS05225; AWH52_RS12700; AWH52_RS22180; AWH52_RS23060; AWH52_RS23535	BR71_RS07305; BR71_RS10435; BR71_RS17900; BR71_RS19420; BR71_RS20430	IEZ30_RS20465	D1345_RS07060; D1345_RS08615; D1345_RS13575; D1345_RS20695; D1345_RS21565	DBB33_07575; DBB33_09690; DBB33_10755; DBB33_13670; DBB33_14575	MOV80_RS01695; MOV80_RS05125; MOV80_RS13335; MOV80_RS15890; MOV80_RS22985	B0T40_RS00460; B0T40_RS08560; B0T40_RS19320; B0T40_RS19910; B0T40_RS23715	CC61_RS00025; CC61_RS02975; CC61_RS11480; CC61_RS20740	GYH29_RS07870; GYH29_RS09125; GYH29_RS14425; GYH29_RS21605; GYH29_RS22630	LF72_RS01130; LF72_RS02075; LF72_RS09950; LF72_RS12890; LF72_RS22690	JD974_RS00110; JD974_RS01075; JD974_RS03265; JD974_RS06560; JD974_RS23430	J1C50_RS00110; J1C50_RS01075; J1C50_RS03265; J1C50_RS06965; J1C50_RS23430
		mtrE	-	-	-	JW897_RS02100	VK98_RS10990	-	-	-	-	-	-	-	-	-	-	IEZ30_RS20470	-	-	-	-	-	-	-	-	-
Immune evasion	Capsule	ctrA	-	-	-	-	-	-	-	-	-	-	-	-	-	-	-	-	-	-	-	-	-	-	-	-	-
		ctrB	-	-	-	-	-	-	-	-	-	-	-	-	-	-	-	-	-	-	-	-	-	-	-	-	-
		ctrC	-	-	-	-	-	-	-	-	-	-	-	-	-	-	-	-	-	-	-	-	-	-	-	-	-
		ctrD	-	-	-	-	-	-	-	-	-	-	-	-	-	-	-	-	-	-	-	-	-	-	-	-	-
		lipA	-	-	-	JW897_RS00265	VK98_RS04690	-	-	JC616_RS22760	HA052_RS00830	B0T39_RS04240	-	-	-	-	-	-	D1345_RS21310	DBB33_10500	MOV80_RS05385	B0T40_RS08815	-	GYH29_RS22370	LF72_RS01380	JD974_RS00815	J1C50_RS00815
		lipB	-	-	-	-	-	-	-	-	-	-	-	-	-	-	-	-	-	-	-	-	-	-	-	-	-
		mynA/sacA	-	-	-	JW897_RS04145	VK98_RS13465	DB032_07490	HQN78_RS05970	JC616_RS22730	HA052_RS07205	B0T39_RS20950	JOS77_RS06800	DXT74_14275	B0T45_RS13355	AWH52_RS14425	BR71_RS01685	IEZ30_RS11465	D1345_RS11550	DBB33_16520	MOV80_RS12180	-	CC61_RS04200	GYH29_RS11920	LF72_RS06365	JD974_RS07655	J1C50_RS09060
		mynB/sacB	-	-	-	-	-	-	-	-	-	-	-	-	-	-	-	-	-	-	-	-	-	-	-	-	-
		mynC/sacC	-	-	-	-	-	-	-	-	-	-	-	-	-	-	-	-	-	-	-	-	-	-	-	-	-
		mynD/sacD	-	-	-	-	-	-	-	-	-	-	-	-	-	-	-	-	-	-	-	-	-	-	-	-	-
		siaA/synA	-	-	-	-	-	-	-	-	-	-	-	-	-	-	-	-	-	-	-	-	-	-	-	-	-
		siaB/synB	-	-	-	-	-	-	-	-	-	-	-	-	-	-	-	-	-	-	-	-	-	-	-	-	-
		siaC/synC	-	-	-	-	-	-	-	-	-	-	-	-	-	-	-	-	-	-	-	-	-	-	-	-	-
		siaD/synD	-	-	-	-	-	-	-	-	-	-	-	-	-	-	-	-	-	-	-	-	-	-	-	-	-
		synE	-	-	-	-	-	-	-	-	-	-	-	-	-	-	-	-	-	-	-	-	-	-	-	-	-
Immune modulator	Factor H binding protein	fHbp	-	-	-	-	-	-	-	-	-	-	-	-	-	-	-	-	-	-	-	-	-	-	-	-	-
	Neisserial surface protein A	nspA	-	-	-	-	-	-	-	-	-	-	-	-	-	-	-	-	-	-	-	-	-	-	-	-	-
Invasion	Class 5 outer membrane protein	opc	-	-	-	-	-	-	-	-	-	-	-	-	-	-	-	-	-	-	-	-	-	-	-	-	-
	Opacity protein	opa	-	-	-	-	-	-	-	-	-	-	-	-	-	-	-	-	-	-	-	-	-	-	-	-	-
	PorA	porA	-	-	-	-	-	-	-	-	-	-	-	-	-	-	-	-	-	-	-	-	-	-	-	-	-
	PorB	porB	-	-	-	-	-	DB032_23485	-	-	-	-	-	-	-	-	-	-	-	-	-	-	-	-	-	-	-
Iron uptake	ABC transporter	fbpA	-	-	-	-	-	-	-	-	-	-	-	-	-	-	-	-	-	-	-	-	-	-	-	-	-
		fbpB	-	-	-	-	-	-	-	-	-	-	-	-	-	-	-	-	-	-	-	-	-	-	-	-	-
		fbpC	-	-	-	-	-	-	-	-	-	-	-	-	-	-	-	-	-	-	-	-	-	-	-	-	-
	Ferric enterobactin transport protein A / ferric-repressed protein B	fetA/frpB	-	-	-	-	-	-	-	-	-	-	-	-	-	-	-	-	-	-	-	-	-	-	-	-	-
	Heme uptake	hpuA	-	-	-	-	-	-	-	-	-	-	-	-	-	-	-	-	-	-	-	-	-	-	-	-	-
		hpuB	-	-	-	-	-	-	-	-	-	-	-	-	-	-	-	-	-	-	-	-	-	-	-	-	-
	Hemoglobin receptor	hmbR	-	-	-	-	-	-	-	-	-	-	-	-	-	-	-	-	-	-	-	-	-	-	-	-	-
	Lactoferrin-binding protein	lbpA	-	-	-	-	-	-	-	-	-	-	-	-	-	-	-	-	-	-	-	-	-	-	-	-	-
		lbpB	-	-	-	-	-	-	-	-	-	-	-	-	-	-	-	-	-	-	-	-	-	-	-	-	-
	Ton system	exbB	MY55_10980	LQT98_RS21330	VI26_RS13010	JW897_RS13245	VK98_RS18040	DB032_22505	HQN78_RS18460	JC616_RS00040	HA052_RS25795	B0T39_RS21530	JOS77_RS04250	DXT74_02785	B0T45_RS18240	AWH52_RS22515	BR71_RS18415	IEZ30_RS23870	D1345_RS23265	DBB33_01050	MOV80_RS17745	B0T40_RS20845	CC61_RS11185	GYH29_RS24195	LF72_RS17990	JD974_RS15810	J1C50_RS16815
		exbD	-	LQT98_RS21325	VI26_RS13005	JW897_RS13250	VK98_RS18045	DB032_22500	HQN78_RS18465	JC616_RS00035	HA052_RS25800	B0T39_RS21525	JOS77_RS04255	DXT74_02790	B0T45_RS18235	AWH52_RS22510	BR71_RS18420	IEZ30_RS23865	D1345_RS23260	DBB33_01045	MOV80_RS17750	B0T40_RS20840	CC61_RS11190	GYH29_RS24190	LF72_RS17995	JD974_RS15805	J1C50_RS16820
		tonB	-	LQT98_RS21335	VI26_RS13015	JW897_RS13235	VK98_RS24255	-	HQN78_RS18455	JC616_RS00045	HA052_RS25790	B0T39_RS21535	JOS77_RS04245	DXT74_02780	B0T45_RS18245	AWH52_RS22520	BR71_RS18410	IEZ30_RS23875	D1345_RS23270	DBB33_01055	MOV80_RS17740	B0T40_RS20850	CC61_RS11180	GYH29_RS24200	LF72_RS17985	JD974_RS15815	J1C50_RS16810
	Transferrin-binding protein	tbpA	-	-	-	-	-	-	-	-	-	-	-	-	-	-	-	-	-	-	-	-	-	-	-	-	-
		tbpB	-	-	-	-	-	-	-	-	-	-	-	-	-	-	-	-	-	-	-	-	-	-	-	-	-
Protease	IgA protease	iga	-	-	-	-	-	-	-	-	-	-	-	-	-	-	-	-	-	-	-	-	-	-	-	-	-
	Zn++ metallophrotease(Mycobacterium)	zmp1	-	LQT98_RS02815	VI26_RS21560	JW897_RS18470	VK98_RS00810	DB032_06345	HQN78_RS08890	JC616_RS13775	HA052_RS14665	B0T39_RS22765	JOS77_RS21520	DXT74_16930	B0T45_RS06675	AWH52_RS23000	BR71_RS00025	IEZ30_RS08215	D1345_RS07955	DBB33_14325	MOV80_RS15240	B0T40_RS00010	CC61_RS10525	GYH29_RS08675	LF72_RS12435	JD974_RS03720	J1C50_RS03720
Stress adaptation	Catalase	katA	MY55_10800	LQT98_RS11030	VI26_RS01920	JW897_RS19910	VK98_RS22385	DB032_23130	HQN78_RS18305	JC616_RS00195	HA052_RS07505	B0T39_RS18775	JOS77_RS10205	DXT74_02635	B0T45_RS18405	AWH52_RS19605	BR71_RS14935	IEZ30_RS24030	D1345_RS23420	DBB33_00765	MOV80_RS18855	B0T40_RS13015	CC61_RS14280	GYH29_RS24350	LF72_RS15530	JD974_RS17280	J1C50_RS17730
	Manganese transport system	mntA	MY55_17925	LQT98_RS02500	VI26_RS00115	JW897_RS20420	VK98_RS15565	DB032_05635	HQN78_RS01160	JC616_RS16640	HA052_RS12465	B0T39_RS20800	JOS77_RS21645	DXT74_09815	B0T45_RS16755	AWH52_RS20140	BR71_RS03985	IEZ30_RS05490	D1345_RS05515	DBB33_15110	MOV80_RS00120	B0T40_RS17115	CC61_RS06965	GYH29_RS16640	LF72_RS21205	JD974_RS20290	J1C50_RS20615
		mntB	MY55_17920	LQT98_RS02495	VI26_RS00120	JW897_RS20425	VK98_RS15570	DB032_05640	HQN78_RS01165	JC616_RS16635	HA052_RS12460	B0T39_RS20795	JOS77_RS21650	DXT74_09820	B0T45_RS16760	AWH52_RS20145	BR71_RS03990	IEZ30_RS05495	D1345_RS05520	DBB33_15105	MOV80_RS00125	B0T40_RS17110	CC61_RS06970	GYH29_RS16635	LF72_RS21200	JD974_RS20285	J1C50_RS20620
		mntC	MY55_17915	LQT98_RS02490	VI26_RS00125	JW897_RS20430	VK98_RS15575	DB032_05645	HQN78_RS01170	JC616_RS16630	HA052_RS12455	B0T39_RS20790	JOS77_RS21655	DXT74_09825	B0T45_RS16765	AWH52_RS20150	BR71_RS03995	IEZ30_RS05500	D1345_RS05525	DBB33_15100	MOV80_RS00130	B0T40_RS17105	CC61_RS06975	GYH29_RS16630	LF72_RS21195	JD974_RS20280	J1C50_RS20625
	Methionine sulphoxide reductase	msrA/B(pilB)	-	-	-	-	-	-	-	-	-	-	-	-	-	-	-	-	-	-	-	-	-	-	-	-	-
	Recombinational repair protein	recN	MY55_07660	LQT98_RS05510	VI26_RS17675	JW897_RS10715	VK98_RS14860	DB032_09440	HQN78_RS07580	JC616_RS10560	HA052_RS03110	B0T39_RS03155	JOS77_RS23375	DXT74_15830	B0T45_RS04100	AWH52_RS08750	BR71_RS20925	IEZ30_RS09400	D1345_RS09550	DBB33_12500	MOV80_RS16600	B0T40_RS01180	-	GYH29_RS09840	LF72_RS18720	JD974_RS02535	J1C50_RS02535
	Catalase-peroxidase(Mycobacterium)	katG	-	LQT98_RS17135	VI26_RS12930	JW897_RS13330	VK98_RS18125	DB032_22410	HQN78_RS18545	JC616_RS24420	HA052_RS20630	B0T39_RS21455	JOS77_RS04315	DXT74_02845	B0T45_RS18175	AWH52_RS19765	BR71_RS18475	IEZ30_RS23795	D1345_RS23190	DBB33_00975	MOV80_RS17820	B0T40_RS20770	CC61_RS17520	GYH29_RS24125	LF72_RS18065	JD974_RS15735	J1C50_RS16890
	SodCI(Salmonella)	sodCI	-	LQT98_RS14195	VI26_RS18475	JW897_RS13130	VK98_RS16510	DB032_03555	HQN78_RS13120	JC616_RS05345	HA052_RS24035	B0T39_RS22860	JOS77_RS10730	DXT74_20480	B0T45_RS00785	AWH52_RS17495	BR71_RS03370	IEZ30_RS04565	D1345_RS04500	DBB33_04090	MOV80_RS02900	B0T40_RS07560	CC61_RS01075	GYH29_RS04470	LF72_RS12015	JD974_RS08975	J1C50_RS09900
Toxin	Neisseria ADP-ribosylating enzyme	narE	-	-	-	-	-	-	-	-	-	-	-	-	-	-	-	-	-	-	-	-	-	-	-	-	-
	RTX toxin	frpA	-	-	-	-	-	-	-	-	-	-	-	-	-	-	-	-	-	-	-	-	-	-	-	-	-
		frpC	-	-	-	-	-	-	-	-	-	-	-	-	-	-	-	-	-	-	-	-	-	-	-	-	-
	Hemolysin HlyA(Aeromonas)	hlyA	MY55_02415	LQT98_RS16810	VI26_RS03010	JW897_RS09055	VK98_RS07775	DB032_00870	HQN78_RS16090	JC616_RS02315	HA052_RS05915	B0T39_RS11855	JOS77_RS02570	DXT74_00845	B0T45_RS07005	AWH52_RS15610	BR71_RS08545	IEZ30_RS01775	D1345_RS01700	DBB33_22960	MOV80_RS10335	B0T40_RS04080	CC61_RS03845	GYH29_RS01690	LF72_RS13645	JD974_RS10445	J1C50_RS11850
	Hydrogen cyanide production(Pseudomonas)	hcnA	MY55_04855	LQT98_RS13595	VI26_RS11240	JW897_RS07195	VK98_RS01735	DB032_08090	HQN78_RS11215	JC616_RS07135	HA052_RS09800	B0T39_RS14280	JOS77_RS18025	DXT74_18895	B0T45_RS17060	AWH52_RS05980	BR71_RS05995	IEZ30_RS05965	D1345_RS05990	DBB33_05185	MOV80_RS00595	B0T40_RS20370	-	GYH29_RS06595	LF72_RS11020	JD974_RS05495	J1C50_RS08030
		hcnB	MY55_04850	LQT98_RS13600	VI26_RS11235	JW897_RS07200	VK98_RS01740	DB032_08085	HQN78_RS11220	JC616_RS07130	HA052_RS09795	B0T39_RS14285	JOS77_RS18030	DXT74_18900	B0T45_RS17055	AWH52_RS05985	BR71_RS06000	IEZ30_RS05960	D1345_RS05985	DBB33_05180	MOV80_RS00590	B0T40_RS20365	-	GYH29_RS06590	LF72_RS11025	JD974_RS05490	J1C50_RS08035
		hcnC	MY55_04845	LQT98_RS13605	VI26_RS11230	JW897_RS07205	VK98_RS01745	DB032_08080	HQN78_RS11225	JC616_RS07125	HA052_RS09790	B0T39_RS14290	JOS77_RS18035	DXT74_18905	B0T45_RS17050	AWH52_RS05990	BR71_RS06005	IEZ30_RS05955	D1345_RS05980	DBB33_05175	MOV80_RS00585	B0T40_RS20360	-	GYH29_RS06585	LF72_RS11030	JD974_RS05485	J1C50_RS08040
Copper uptake	Copper exporter(Mycobacterium)	ctpV	MY55_20950	-	-	JW897_RS15165	VK98_RS02335	DB032_04620	HQN78_RS00195	JC616_RS17860	HA052_RS11375	B0T39_RS21800	-	-	-	-	-	-	D1345_RS17280	DBB33_19760	MOV80_RS11735	B0T40_RS08525	CC61_RS15575	GYH29_RS17675	LF72_RS07640	JD974_RS20410	J1C50_RS21235
Enzyme	Non-hemolytic phospholipase C(Pseudomonas)	plcN	-	-	VI26_RS06445	JW897_RS11365	VK98_RS17275	DB032_07840	HQN78_RS06860	JC616_RS11305	HA052_RS03800	B0T39_RS02415	JOS77_RS08310	DXT74_15125	B0T45_RS13735	AWH52_RS05705	BR71_RS06645	IEZ30_RS10055	D1345_RS10225	DBB33_07365	MOV80_RS14405	B0T40_RS23225	CC61_RS04780	GYH29_RS10550	LF72_RS17505	JD974_RS12800	J1C50_RS13380
Lipid and fatty acid metabolism	Isocitrate lyase(Mycobacterium)	icl	MY55_04630	LQT98_RS15315	VI26_RS18290	JW897_RS17550	VK98_RS16320	DB032_03755	HQN78_RS12740	JC616_RS05535	HA052_RS06510	B0T39_RS23670	JOS77_RS10795	DXT74_20415	B0T45_RS00720	AWH52_RS17430	BR71_RS03430	IEZ30_RS04680	D1345_RS04615	DBB33_18425	MOV80_RS02705	B0T40_RS13820	CC61_RS06335	GYH29_RS04665	LF72_RS11790	JD974_RS08740	J1C50_RS10135
	Pantothenate synthesis(Mycobacterium)	panD	MY55_04595	LQT98_RS18355	VI26_RS01080	JW897_RS06135	VK98_RS09775					B0T39_RS07610	-	-	-	-	-	-	-	-	-	-	-	-	LF72_RS10245	JD974_RS06265	J1C50_RS07260
Macrophage inducible genes	Mig-5(Salmonella)	mig-5	-	LQT98_RS17660	VI26_RS21180	JW897_RS21980	VK98_RS13000	DB032_01945	-	JC616_RS03565	HA052_RS21925	B0T39_RS15935	JOS77_RS18985	DXT74_22130		AWH52_RS10935	BR71_RS12980	IEZ30_RS02955	D1345_RS02890	DBB33_00005	MOV80_RS22045	B0T40_RS15335	CC61_RS09065	GYH29_RS02880	LF72_RS20965	JD974_RS19380	J1C50_RS20110
Nutritional virulence	Cysteine acquisition(Francisella)		-	LQT98_RS13455	VI26_RS20250	JW897_RS03915	VK98_RS17840	DB032_08315	HQN78_RS06280	JC616_RS11960	HA052_RS06965	B0T39_RS20165	JOS77_RS06970	DXT74_14445	B0T45_RS13185	AWH52_RS09905	BR71_RS01855	IEZ30_RS11205	D1345_RS11310	DBB33_06660	MOV80_RS15115	B0T40_RS06105	CC61_RS00140	GYH29_RS11690	LF72_RS06600	JD974_RS07885	J1C50_RS09290
Regulation	Alternative sigma factor RpoS(Legionella)	rpoS	MY55_10205	LQT98_RS16090	VI26_RS08565	JW897_RS11965	VK98_RS19800	DB032_02690	HQN78_RS14030	JC616_RS04370	HA052_RS02040	B0T39_RS00270	JOS77_RS03220	DXT74_21360	B0T45_RS01665	AWH52_RS00295	BR71_RS02455	IEZ30_RS03690	D1345_RS03620	DBB33_19865	MOV80_RS03775	B0T40_RS06695	CC61_RS16280	GYH29_RS03620	LF72_RS16105	JD974_RS16835	J1C50_RS15650
Serum resistance	PbpG(Acinetobacter)	pbpG	-	-	-	-	-	DB032_11860	HQN78_RS04335	JC616_RS09135	HA052_RS22405	B0T39_RS22725	-	-	-	-	-	IEZ30_RS13120	D1345_RS13150	DBB33_16375	MOV80_RS17635	B0T40_RS09575	CC61_RS21895	GYH29_RS14000	LF72_RS16720	JD974_RS15085	J1C50_RS16640

Table S2. Bacterial strains used in the study.

Genus	Species	Strain	GenBank Accession	Phylum	Class	Order	Family	Collection Source
Microbacterium		Lab0001	KC192341	Actinobacteria	Actinobacteria	Actinomycetales	Microbacteriaceae	CN laboratory culture
Sphingobacterium		Lab0004	MN544616	Bacteroidetes	Sphingobacteria	Sphingobacteriales	Sphingobacteriaceae	CN laboratory culture
Flectobacillus		Rain0001	MN544619	Bacteroidetes	Cytophagia	Cytophagales	Cytophagaceae	CN field culture
Rahnella		Rain0009	MN544617	Proteobacteria	Gammaproteobacteria	Enterobacteriales	Enterobacteriacae	CN field culture
Serratia		Rain0010	MN544614	Proteobacteria	Gammaproteobacteria	Enterobacteriales	Enterobacteriacae	CN field culture
Acinetobacter		River0008	MN544618	Proteobacteria	Gammaproteobacteria	Pseudomonadales	Moraxellaceae	CN field culture
Chryseobacterium		Lab	KC192341	Bacteroidota	Flavobacterila	Flavobacteriales	Weeksellaceae	CN laboratory culture
Comamonas		Lab	KX260133	Pseudomonadota	Betaproteobacteria	Burkholderiales	Comamonadaceae	CN laboratory culture
Escherichia	*coli*	K-12 substr. MG1655	U00096	Proteobacteria	Gammaproteobacteria	Enterobacteriales	Enterobacteriacae	Lab Strain
Chromobacterium	*haemolyticum*	*Ch_* R13	GCA_015291765.1	Pseudomonadota	Pseudomonadota	Neisseriales	Neisseriaceae	0013 container (this study)
Chromobacterium	*haemolyticum*	DSM 19808	GCA_000711885.1	Pseudomonadota	Pseudomonadota	Neisseriales	Neisseriaceae	Leibniz Inst., Germany
Chromobacterium	*haemolyticum*	NRRL B-11053	GCA_003052545.1	Pseudomonadota	Pseudomonadota	Neisseriales	Neisseriaceae	US Dept. Agriculture

Table S3. Multivariate binomial regression analysis of the percentage of larvae that pupated in the data set presented in Fig. 4. Replicate was included as a random effect for the model that examined individual species effects and the model that examined 2-species interactions. In both cases, the estimated among-replicate variance was very small (0.000217 and 0.001592, respectively) when compared to the magnitude of any treatment effects.

Model	Parameter	Estimate	Std. Error	*z* Value	Pr(>|*z*|)
Individual species effects	Intercept	-2.96709	0.06909	-42.945	<2E-16
	chryseo	1.21498	0.05707	21.29	<2E-16
	acine	1.74084	0.05816	29.933	<2E-16
	ecoli	0.65145	0.06515	9.999	<2E-16
	micro	0.6363	0.05934	10.723	<2E-16
	coma	0.92443	0.06029	15.334	<2E-16
	sphingo	1.51921	0.05907	25.718	<2E-16
	rahnella	-0.31775	0.06948	-4.573	4.8E-06
	serr	0.19101	0.06526	2.927	0.00342
	flecto	0.53322	0.05759	9.259	<2E-16
Species interactions	Intercept	-4.5479	0.20569	-22.111	<2E-16
	chryseo	2.62358	0.17145	15.302	<2E-16
	acine	2.71187	0.16121	16.822	<2E-16
	ecoli	1.16763	0.17556	6.651	2.91E-11
	micro	1.36612	0.17978	7.599	2.99E-14
	coma	2.22648	0.18339	12.14	<2E-16
	sphingo	2.98401	0.16584	17.993	<2E-16
	rahnella	-0.96989	0.20437	-4.746	2.08E-06
	serr	0.08475	0.19627	0.432	0.665869
	flecto	0.34524	0.1722	2.005	0.044980
	chryseo:acine	1.25793	0.1535	8.195	2.50E-16
	chryseo:ecoli	-0.36756	0.17895	-2.054	0.039976
	chryseo:micro	-1.31421	0.14957	-8.787	<2E-16
	chryseo:coma	-1.69523	0.15721	-10.783	<2E-16
	chryseo:sphingo	-1.99207	0.14619	-13.626	<2E-16
	chryseo:rahnella	1.68173	0.22145	7.594	3.09E-14
	chryseo:serr	-0.13667	0.1972	-0.693	0.488284
	chryseo:flecto	-0.39167	0.13958	-2.806	0.005014
	acine:ecoli	-0.06391	0.17799	-0.359	0.719545
	acine:micro	-0.96451	0.14872	-6.485	8.85E-11
	acine:coma	-1.50922	0.15079	-10.009	<2E-16
	acine:sphingo	-0.25074	0.14129	-1.775	0.075960
	acine:rahnella	-0.95715	0.21002	-4.557	5.18E-06
	acine:serr	0.53361	0.19068	2.798	0.005135
	acine:flecto	0.0545	0.14132	0.386	0.699735
	ecoli:micro	-1.36749	0.20759	-6.587	4.48E-11
	ecoli:coma	-0.23789	0.17048	-1.395	0.162892
	ecoli:sphingo	1.28407	0.20101	6.388	1.68E-10
	ecoli:rahnella	-0.43156	0.19037	-2.267	0.023391
	ecoli:serr	0.37311	0.18339	2.034	0.041903
	ecoli:flecto	0.16034	0.17372	0.923	0.356025
	micro:coma	0.38842	0.15385	2.525	0.011580
	micro:sphingo	-0.1375	0.15309	-0.898	0.369108
	micro:rahnella	1.37592	0.21879	6.289	3.20E-10
	micro:serr	0.22247	0.18509	1.202	0.229376
	micro:flecto	0.36617	0.14804	2.473	0.013385
	coma:sphingo	0.02362	0.15443	0.153	0.878423
	coma:rahnella	0.95188	0.20255	4.7	2.61E-06
	coma:serr	-0.02083	0.175	-0.119	0.905236
	coma:flecto	-0.09064	0.15859	-0.572	0.567627
	sphingo:rahnella	-0.73568	0.20354	-3.614	0.000301
	sphingo:serr	-0.621	0.179	-3.469	0.000522
	sphingo:flecto	0.11979	0.14731	0.813	0.416126
	rahnella:serr	-0.60983	0.2007	-3.038	0.002378
	rahnella:flecto	-0.68891	0.18812	-3.662	0.000250
	serr:flecto	0.95313	0.17177	5.549	2.88E-08

Table S4. Multivariate binomial regression analysis of the percentage of larvae that survived to adulthood in the data set presented in Fig. 4. Replicate was removed as a random effect for both models due to the estimated among-replicate variance approximating zero.

Model	Parameter	Estimate	Std. Error	*z* Value	Pr(>|*z*|)
Individual species effects	(Intercept)	-3.97822	0.08209	-48.464	<2E-16
	chryseo	1.12691	0.0559	20.16	<2E-16
	acine	1.24418	0.05661	21.98	<2E-16
	ecoli	0.29979	0.0592	5.064	4.11E-07
	micro	0.52889	0.05565	9.505	<2E-16
	coma	0.76481	0.05324	14.365	<2E-16
	sphingo	0.76021	0.05657	13.438	<2E-16
	rahnella	0.36085	0.05949	6.066	1.31E-09
	serr	0.32697	0.05826	5.612	2.00E-08
	flecto	0.35059	0.05449	6.434	1.25E-10
Species interactions	Intercept	-6.92586	0.28866	-23.993	<2E-16
	chryseo	1.43494	0.23595	6.082	1.19E-09
	acine	3.35445	0.24795	13.529	<2E-16
	ecoli	1.41708	0.23912	5.926	3.10E-09
	micro	1.05866	0.23459	4.513	6.40E-06
	coma	2.28543	0.22806	10.021	<2E-16
	sphingo	3.05393	0.23867	12.796	<2E-16
	rahnella	-0.06587	0.24142	-0.273	0.784965
	serr	0.05346	0.24616	0.217	0.828086
	flecto	0.62865	0.22957	2.738	0.006174
	chryseo:acine	1.37089	0.14736	9.303	<2E-16
	chryseo:ecoli	0.02141	0.17346	0.123	0.901784
	chryseo:micro	-0.10159	0.15443	-0.658	0.510670
	chryseo:coma	-0.61931	0.15425	-4.015	5.95E-05
	chryseo:sphingo	-1.70323	0.17296	-9.848	<2E-16
	chryseo:rahnella	0.74265	0.17761	4.181	2.90E-05
	chryseo:serr	0.04495	0.17488	0.257	0.797162
	chryseo:flecto	-0.09405	0.15056	-0.625	0.532184
	acine:ecoli	-0.65628	0.18072	-3.631	0.000282
	acine:micro	-0.67882	0.16438	-4.13	3.63E-05
	acine:coma	-1.4026	0.16129	-8.696	<2E-16
	acine:sphingo	-0.77432	0.18187	-4.257	2.07E-05
	acine:rahnella	-0.26698	0.1824	-1.464	0.143260
	acine:serr	-0.06196	0.18047	-0.343	0.731341
	acine:flecto	-0.47482	0.16117	-2.946	0.003218
	ecoli:micro	0.0829	0.16956	0.489	0.624913
	ecoli:coma	-0.48064	0.14183	-3.389	0.000702
	ecoli:sphingo	-0.27385	0.17966	-1.524	0.12744
	ecoli:rahnella	0.218	0.14946	1.459	0.144678
	ecoli:serr	-0.33008	0.14945	-2.209	0.027201
	ecoli:flecto	-0.12741	0.15177	-0.839	0.4012
	micro:coma	0.14972	0.13983	1.071	0.284288
	micro:sphingo	-0.15695	0.15515	-1.012	0.311721
	micro:rahnella	-0.32475	0.16059	-2.022	0.043145
	micro:serr	0.0781	0.15881	0.492	0.622858
	micro:flecto	0.22831	0.14065	1.623	0.104547
	coma:sphingo	-0.38093	0.15071	-2.528	0.011484
	coma:rahnella	0.98618	0.15217	6.481	9.13E-11
	coma:serr	-0.24099	0.13921	-1.731	0.083433
	coma:flecto	0.03164	0.13695	0.231	0.817295
	sphingo:rahnella	-0.3186	0.17265	-1.845	0.064994
	sphingo:serr	0.50926	0.17299	2.944	0.003241
	sphingo:flecto	0.07262	0.15414	0.471	0.637578
	rahnella:serr	-0.04199	0.15235	-0.276	0.782855
	rahnella:flecto	-0.49112	0.15417	-3.186	0.001445
	serr:flecto	0.43575	0.14712	2.962	0.003058

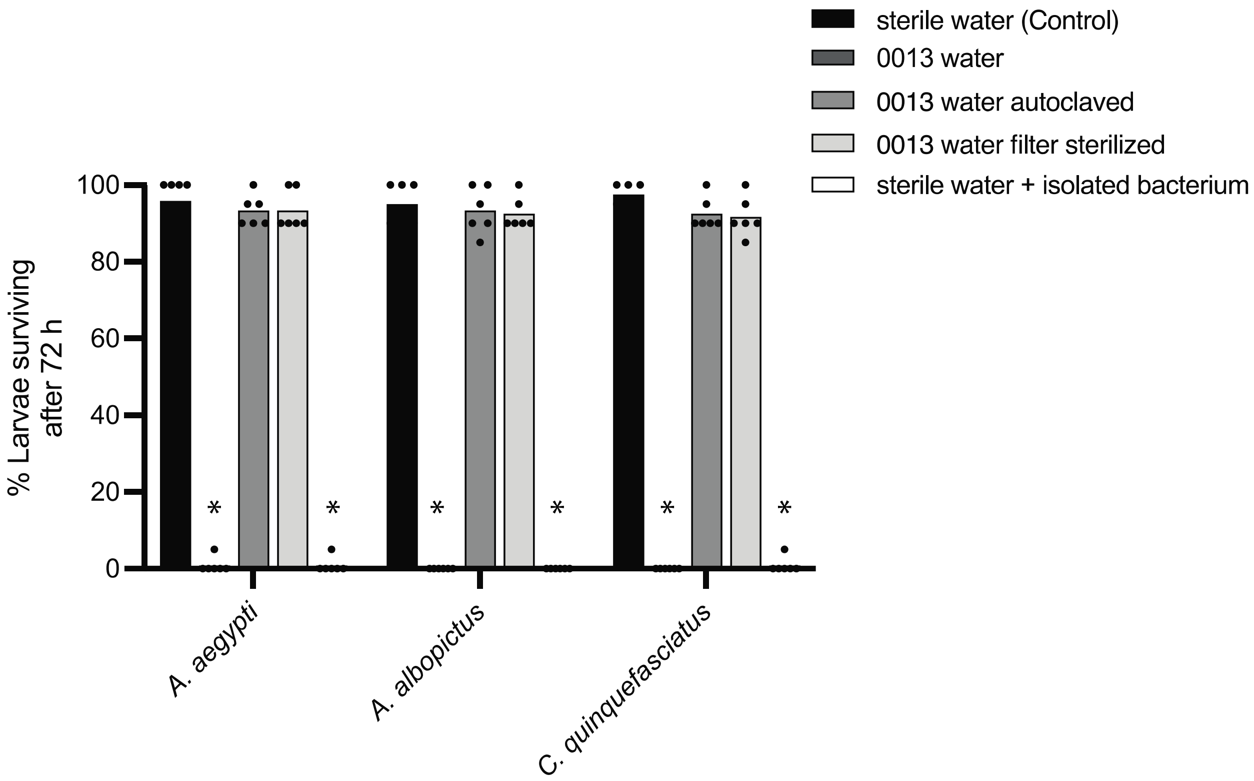

Fig S1 Percentage of CN *A. aegypti, A. albopictus,* or *C. quinquefasciatus* first instars that were alive 72 h after placement in sterile water (Control), 0013 water, 0013 water that was autoclaved, 0013 water that was sterilized through an 0.2 um filter, or sterile water plus the larvicidal bacterium that was isolated from 0013 water. Each replicate is a culture well with 10 larvae (6 replicates per treatment. Columns show mean values for each treatment with each replicate shown as solid circles. For each mosquito species, an asterisk (*) indicates a significant difference for a given treatment when compared to the Control as determined by a Fisher's exact test (P<0.0001).

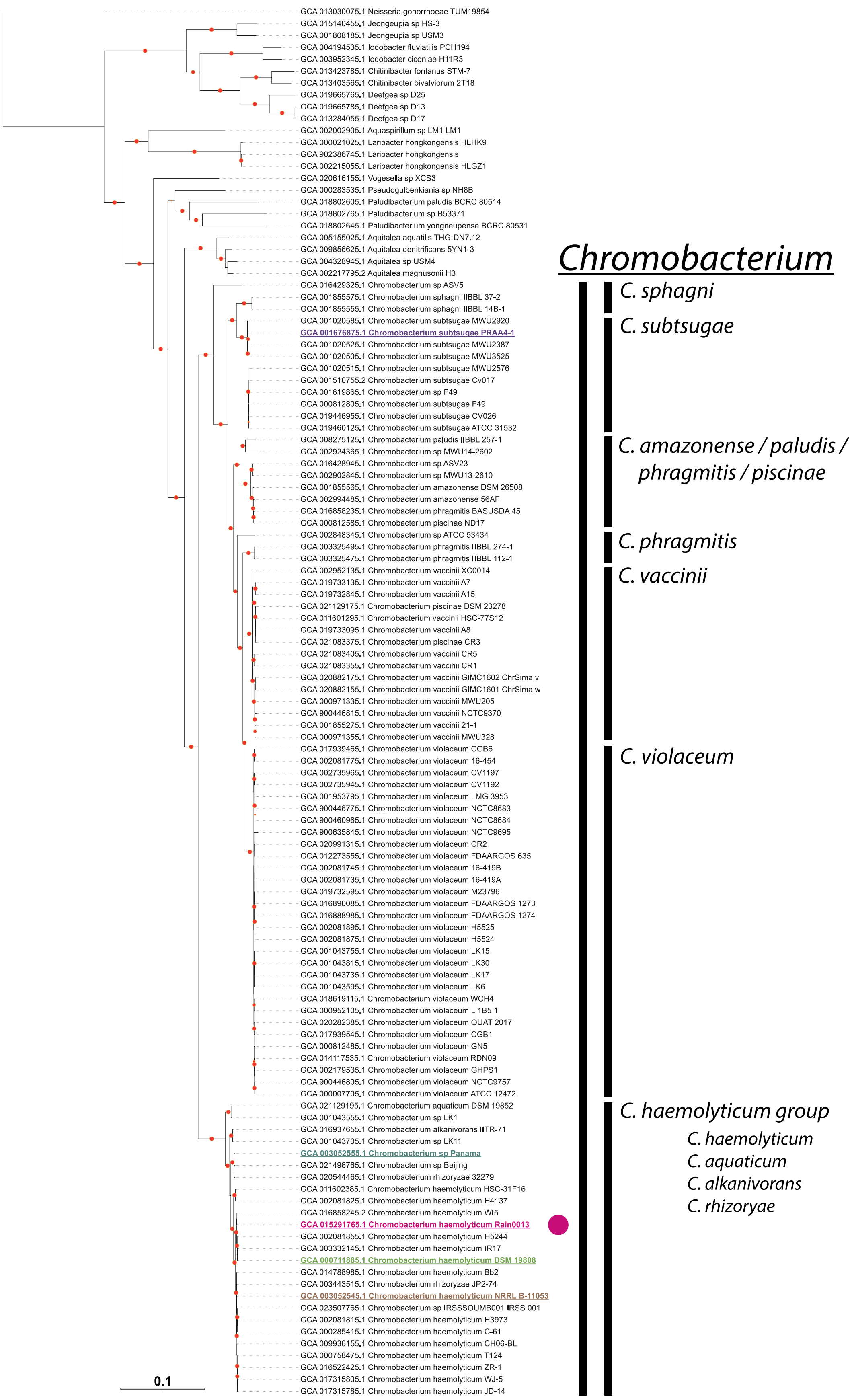

GCA 013030075.1 Neisseria gonorrhoeae TUM19854
GCA 015140455.1 Jeongeupia sp HS-3
GCA 001808185.1 Jeongeupia sp USM3
GCA 004194535.1 Iodobacter fluviatilis PCH194
GCA 003952345.1 Iodobacter ciconiae H11R3
GCA 013423785.1 Chitinibacter fontanus STM-7
GCA 013403565.1 Chitinibacter bivalviorum 2T18
GCA 019665765.1 Deefgea sp D25
GCA 019665785.1 Deefgea sp D13
GCA 013284055.1 Deefgea sp D17
GCA 002002905.1 Aquaspirillum sp LM1 LM1
GCA 000021025.1 Laribacter hongkongensis HLHK9
GCA 902386745.1 Laribacter hongkongensis
GCA 002215055.1 Laribacter hongkongensis HLGZ1
GCA 020616155.1 Vogesella sp XCS3
GCA 000283535.1 Pseudogulbenkiania sp NH8B
GCA 018802605.1 Paludibacterium paludis BCRC 80514
GCA 018802765.1 Paludibacterium sp B53371
GCA 018802645.1 Paludibacterium yongneupense BCRC 80531
GCA 005155025.1 Aquitalea aquatilis THG-DN7.12
GCA 009856625.1 Aquitalea denitrificans 5YN1-3
GCA 004328945.1 Aquitalea sp USM4
GCA 002217795.2 Aquitalea magnusonii H3
GCA 016429325.1 Chromobacterium sp ASV5
GCA 001855575.1 Chromobacterium sphagni IIBBL 37-2
GCA 001855555.1 Chromobacterium sphagni IIBBL 14B-1
GCA 001020585.1 Chromobacterium subtsugae MWU2920
GCA 001676875.1 Chromobacterium subtsugae PRAA4-1
GCA 001020525.1 Chromobacterium subtsugae MWU2387
GCA 001020505.1 Chromobacterium subtsugae MWU3525
GCA 001020515.1 Chromobacterium subtsugae MWU2576
GCA 001510755.2 Chromobacterium subtsugae Cv017
GCA 001619865.1 Chromobacterium sp F49
GCA 000812805.1 Chromobacterium subtsugae F49
GCA 019446955.1 Chromobacterium subtsugae CV026
GCA 019460125.1 Chromobacterium subtsugae ATCC 31532
GCA 008275125.1 Chromobacterium paludis IIBBL 257-1
GCA 002924365.1 Chromobacterium sp MWU14-2602
GCA 016428945.1 Chromobacterium sp ASV23
GCA 002902845.1 Chromobacterium sp MWU13-2610
GCA 001855565.1 Chromobacterium amazonense DSM 26508
GCA 002994485.1 Chromobacterium amazonense 56AF
GCA 016858235.1 Chromobacterium phragmitis BASUSDA 45
GCA 000812585.1 Chromobacterium piscinae ND17
GCA 002848345.1 Chromobacterium sp ATCC 53434
GCA 003325495.1 Chromobacterium phragmitis IIBBL 274-1
GCA 003325475.1 Chromobacterium phragmitis IIBBL 112-1
GCA 002952135.1 Chromobacterium vaccinii XC0014
GCA 019733135.1 Chromobacterium vaccinii A7
GCA 019732845.1 Chromobacterium vaccinii A15
GCA 021129175.1 Chromobacterium piscinae DSM 23278
GCA 011601295.1 Chromobacterium vaccinii HSC-77S12
GCA 019733095.1 Chromobacterium vaccinii A8
GCA 021083375.1 Chromobacterium piscinae CR3
GCA 021083405.1 Chromobacterium vaccinii CR5
GCA 021083355.1 Chromobacterium vaccinii CR1
GCA 020882175.1 Chromobacterium vaccinii GIMC1602 ChrSima v
GCA 020882155.1 Chromobacterium vaccinii GIMC1601 ChrSima w
GCA 000971335.1 Chromobacterium vaccinii MWU205
GCA 900446815.1 Chromobacterium vaccinii NCTC9370
GCA 001855275.1 Chromobacterium vaccinii 21-1
GCA 000971355.1 Chromobacterium vaccinii MWU328
GCA 017939465.1 Chromobacterium violaceum CGB6
GCA 002081775.1 Chromobacterium violaceum 16-454
GCA 002735965.1 Chromobacterium violaceum CV1197
GCA 002735945.1 Chromobacterium violaceum CV1192
GCA 001953795.1 Chromobacterium violaceum LMG 3953
GCA 900446775.1 Chromobacterium violaceum NCTC8683
GCA 900460965.1 Chromobacterium violaceum NCTC8684
GCA 900635845.1 Chromobacterium violaceum NCTC9695
GCA 020991315.1 Chromobacterium violaceum CR2
GCA 012273555.1 Chromobacterium violaceum FDAARGOS 635
GCA 002081745.1 Chromobacterium violaceum 16-419B
GCA 002081735.1 Chromobacterium violaceum 16-419A
GCA 019732595.1 Chromobacterium violaceum M23796
GCA 016890085.1 Chromobacterium violaceum FDAARGOS 1273
GCA 016888985.1 Chromobacterium violaceum FDAARGOS 1274
GCA 002081895.1 Chromobacterium violaceum H5525
GCA 002081875.1 Chromobacterium violaceum H5524
GCA 001043755.1 Chromobacterium violaceum LK15
GCA 001043815.1 Chromobacterium violaceum LK30
GCA 001043735.1 Chromobacterium violaceum LK17
GCA 001043595.1 Chromobacterium violaceum LK6
GCA 018619115.1 Chromobacterium violaceum WCH4
GCA 000952105.1 Chromobacterium violaceum L 1B5 1
GCA 020282385.1 Chromobacterium violaceum OUAT 2017
GCA 017939545.1 Chromobacterium violaceum CGB1
GCA 000812485.1 Chromobacterium violaceum GN5
GCA 014117535.1 Chromobacterium violaceum RDN09
GCA 002179535.1 Chromobacterium violaceum GHPS1
GCA 900446805.1 Chromobacterium violaceum NCTC9757
GCA 000007705.1 Chromobacterium violaceum ATCC 12472
GCA 021129195.1 Chromobacterium aquaticum DSM 19852
GCA 001043555.1 Chromobacterium sp LK1
GCA 016937655.1 Chromobacterium alkanivorans IITR-71
GCA 001043705.1 Chromobacterium sp LK11
GCA 003052555.1 Chromobacterium sp Panama
GCA 021496765.1 Chromobacterium sp Beijing
GCA 020544465.1 Chromobacterium rhizoryzae 32279
GCA 011602385.1 Chromobacterium haemolyticum HSC-31F16
GCA 002081825.1 Chromobacterium haemolyticum H4137
GCA 016858245.2 Chromobacterium haemolyticum WI5
GCA 015291785.1 Chromobacterium haemolyticum Rain0013
GCA 002081855.1 Chromobacterium haemolyticum H5244
GCA 003332145.1 Chromobacterium haemolyticum IR17
GCA 000711885.1 Chromobacterium haemolyticum DSM 19808
GCA 014788985.1 Chromobacterium haemolyticum Bb2
GCA 003443515.1 Chromobacterium rhizoryzae JP2-74
GCA 003052545.1 Chromobacterium haemolyticum NRRL B-11053
GCA 023507765.1 Chromobacterium sp IRSSSOUMB001 IRSS 001
GCA 002081815.1 Chromobacterium haemolyticum H3973
GCA 000285415.1 Chromobacterium haemolyticum C-61
GCA 009936155.1 Chromobacterium haemolyticum CH06-BL
GCA 000758475.1 Chromobacterium haemolyticum T124
GCA 016522425.1 Chromobacterium haemolyticum ZR-1
GCA 017315805.1 Chromobacterium haemolyticum WJ-5
GCA 017315785.1 Chromobacterium haemolyticum JD-14
0.1
Chromobacterium
C. sphagni
C. subtsugae
C. amazonense / paludis / phragmitis / piscinae
C. phragmitis
C. vaccinii
C. violaceum
C. haemolyticum group
C. haemolyticum
C. aquaticum
C. alkanivorans
C. rhizoryae

Fig S2 Phylogenetic tree for the genus *Chromobacterium* visualized in iTOL (red circles indicate full SH-like support) using 203 single-copy orthologous genes in GoToTree with the default settings for all publicly available genomes (FastTree2; scale bar = substitutions per site). Select species of closely related genera are also shown. *Chromobacterium* spp. supported by the phylogeny are indicated to the right. Strong support for the *C. haemolyticum* group suggests *C. aquaticum*, *C. alkanivorans*, and *C. rhizoryae* are strains of *C. haemolyticum*.

FastANI ANI values (clustered by ANI)

Group	Strain	Accession	GCA_016858245.2	GCA_015291765.1	GCA_000711885.1	GCA_003052545.1	GCA_014788985.1	GCA_009936155.1	GCA_021496765.1	GCA_020544465.1	GCA_003052555.1	GCA_016937655.1	GCA_011602385.1	GCA_021129195.1
C. haemolyticum	WI5	GCA_016858245.2	100	98.58	98.48	95.99	95.9	95.95	91.8	91.8	91.69	91.88	93.69	89.63
	Rain0013	GCA_015291765.1	98.59	100	98.65	96.11	96.01	96.22	91.79	91.9	91.84	91.96	93.9	89.82
	DSM 19808	GCA_000711885.1	98.5	98.63	100	96.2	96.01	96.13	91.72	91.76	91.71	91.94	93.92	89.63
	NRRL B-11053	GCA_003052545.1	95.98	96.13	96.16	100	98.58	98.47	92.23	92.23	92.2	92.89	94.02	89.86
	Bb2	GCA_014788985.1	95.89	96.1	96.08	98.59	100	98.76	92.37	92.33	92.31	92.97	94.04	90.01
	CH06-BL	GCA_009936155.1	95.94	96.23	96.06	98.55	98.76	100	92.2	92.22	92.16	92.82	94.01	89.74
C. rhizoryzae	Beijing	GCA_021496765.1	91.77	91.82	91.76	92.27	92.31	92.17	100	98.54	98.5	93.61	93.43	91.84
	32279	GCA_020544465.1	91.76	91.82	91.74	92.19	92.27	92.25	98.55	100	98.53	93.67	93.45	91.65
	Panama	GCA_003052555.1	91.67	91.79	91.75	92.16	92.28	92.14	98.45	98.45	100	93.59	93.45	91.84
C. alkanivorans	IITR-71	GCA_016937655.1	91.79	91.99	91.97	92.83	92.99	92.86	93.63	93.64	93.68	100	92.57	91.06
***C. haemolyticum* 2**	HSC-31F16	GCA_011602385.1	93.73	93.96	93.85	94.01	94	93.97	93.46	93.44	93.44	92.6	100	90.29
C. aquaticum	DSM 19852	GCA_021129195.1	89.59	89.77	89.67	89.9	89.96	89.84	91.74	91.76	91.77	91.17	90.34	100

Fig S3 Average nucleotide identity (ANI) analysis. The *C. haemolyticum, C. rhizoryzae, C. alkanivorans, C. haemolyticum 2,* and *C. aquaticum* groups are indicated to the left of the matrix with strains in each group and associated accession numbers. The matrix to the right presents ANI values for each pairwise comparison of strains with values ≥95% highlighted in dark green, light green and yellow boxes.

A

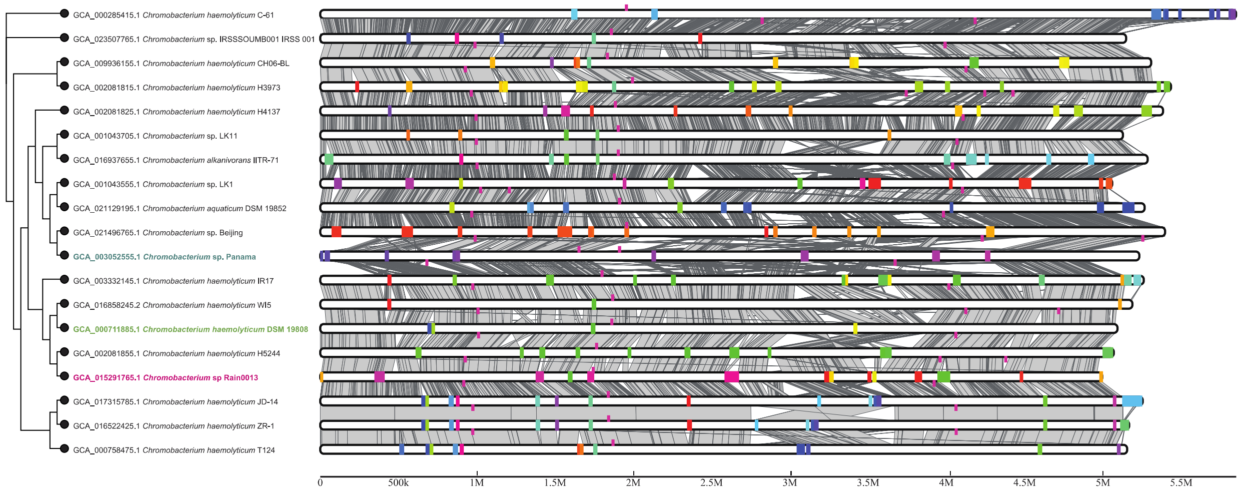

B

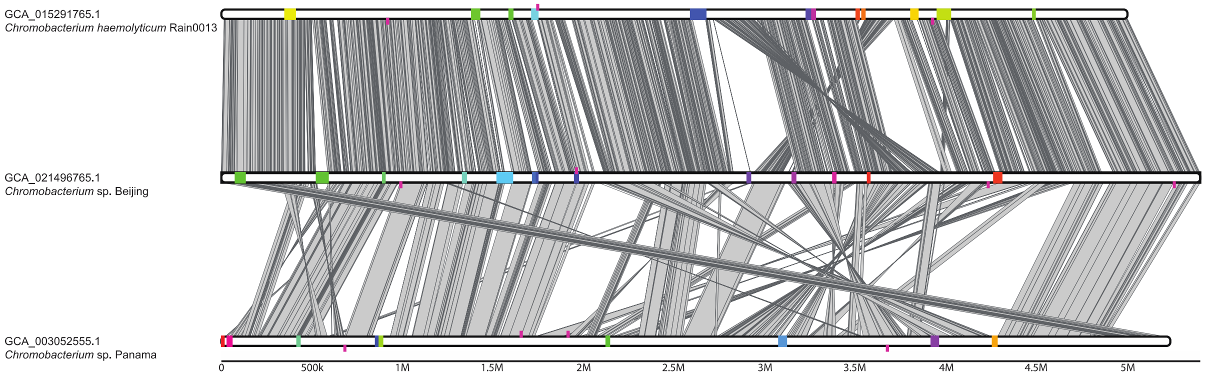

Fig S4 Genomic synteny among members of the *C. haemolyticum* group. (A) Dendrogram of species clustered by genome synteny similarity between 19 genomes in the *C. haemolyticum* group. Chromosomes are indicated by the white bars with genomic islands designated by different color regions. Similar colors represent similar genomic islands. Syntenic regions between genomes are linked by light gray with dark gray edges to indicate breaks in synteny. Pink ticks above or below the chromosomes represent antimicrobial resistance-associated genes. Genomes were rearranged to the *C. haemolyticum* Rain0013 isolate and the Island Compare

webserver was used to assess synteny and draw figure. (B) Close up view of the data in (A) that compares genome synteny of *C. haemolyticum* Rain0013 to *C.* sp. Beijing and *C.* sp. Panama.

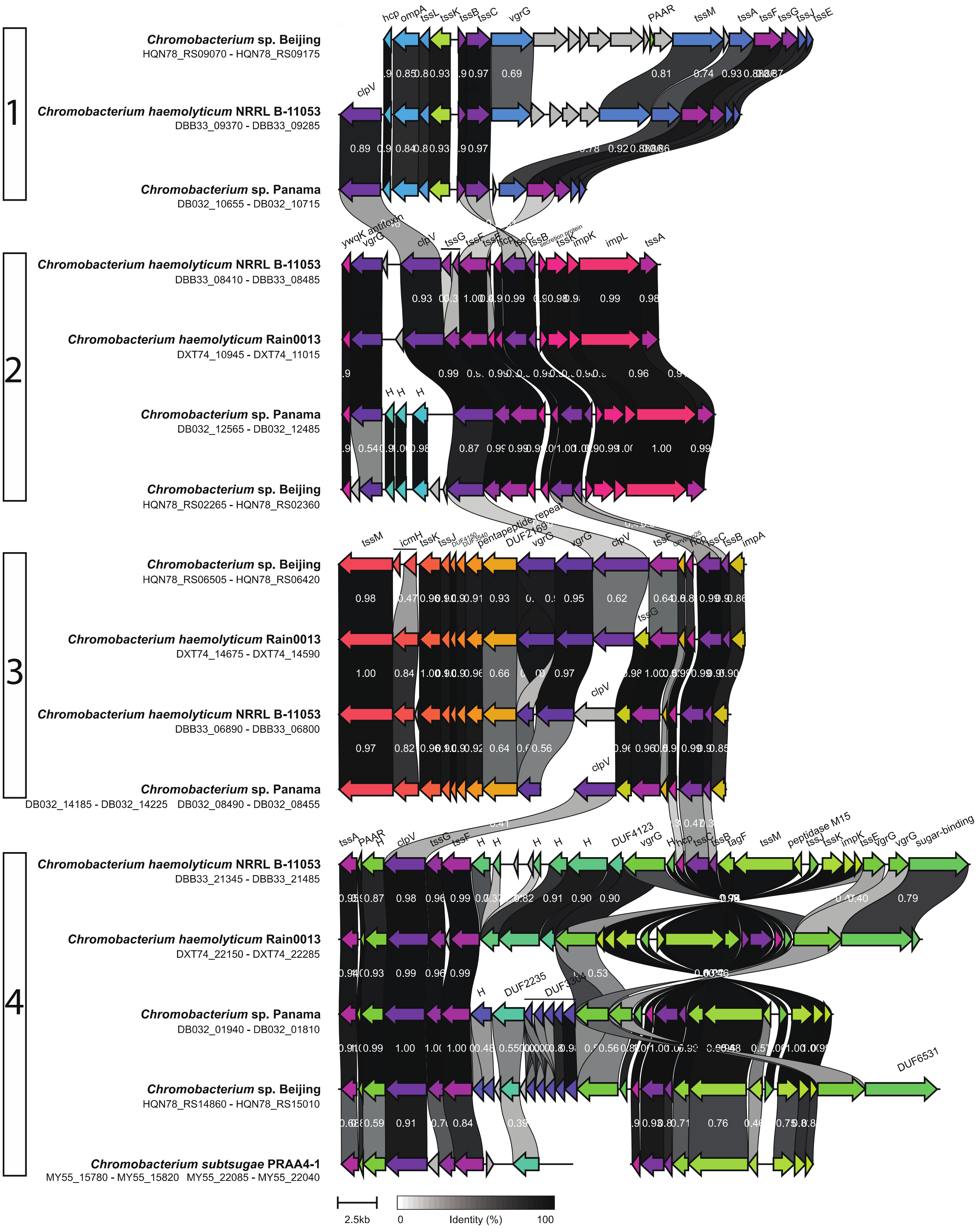
1
Chromobacterium sp. Beijing
HQN78_RS09070 - HQN78_RS09175
Chromobacterium haemolyticum NRRL B-11053
DBB33_09370 - DBB33_09285
Chromobacterium sp. Panama
DB032_10655 - DB032_10715
2
Chromobacterium haemolyticum NRRL B-11053
DBB33_08410 - DBB33_08485
Chromobacterium haemolyticum Rain0013
DXT74_10945 - DXT74_11015
Chromobacterium sp. Panama
DB032_12565 - DB032_12485
Chromobacterium sp. Beijing
HQN78_RS02265 - HQN78_RS02360
3
Chromobacterium sp. Beijing
HQN78_RS06505 - HQN78_RS06420
Chromobacterium haemolyticum Rain0013
DXT74_14675 - DXT74_14590
Chromobacterium haemolyticum NRRL B-11053
DBB33_06890 - DBB33_06800
Chromobacterium sp. Panama
DB032_14185 - DB032_14225 DB032_08490 - DB032_08455
4
Chromobacterium haemolyticum NRRL B-11053
DBB33_21345 - DBB33_21485
Chromobacterium haemolyticum Rain0013
DXT74_22150 - DXT74_22285
Chromobacterium sp. Panama
DB032_01940 - DB032_01810
Chromobacterium sp. Beijing
HQN78_RS14860 - HQN78_RS15010
Chromobacterium subtsugae PRAA4-1
MY55_15780 - MY55_15820 MY55_22085 - MY55_22040
2.5kb
0
Identity (%)
100

Fig S5 Comparative analysis of type VI secretion system (T6SS) operons in five *Chromobacterium* genomes: *C. haemolyticum* Rain0013, *C. haemolyticum* NRRL_B-11053, *C.* sp. Panama, *C.* sp. Beijing, and *C. subtsugae* PRAA4-1. Homologous genes are colored similarly and are connected by shaded links corresponding to sequence identity (black = 100%, white = 0%). Singleton genes colored gray were annotated as hypothetical proteins (H) unless labeled otherwise. Major components of the T6SS operon are labeled according to Table 1 of Shyntum et al. (1). Locus tags for genes included in each T6SS operon are listed, although some operons span gaps in the genome assembly.

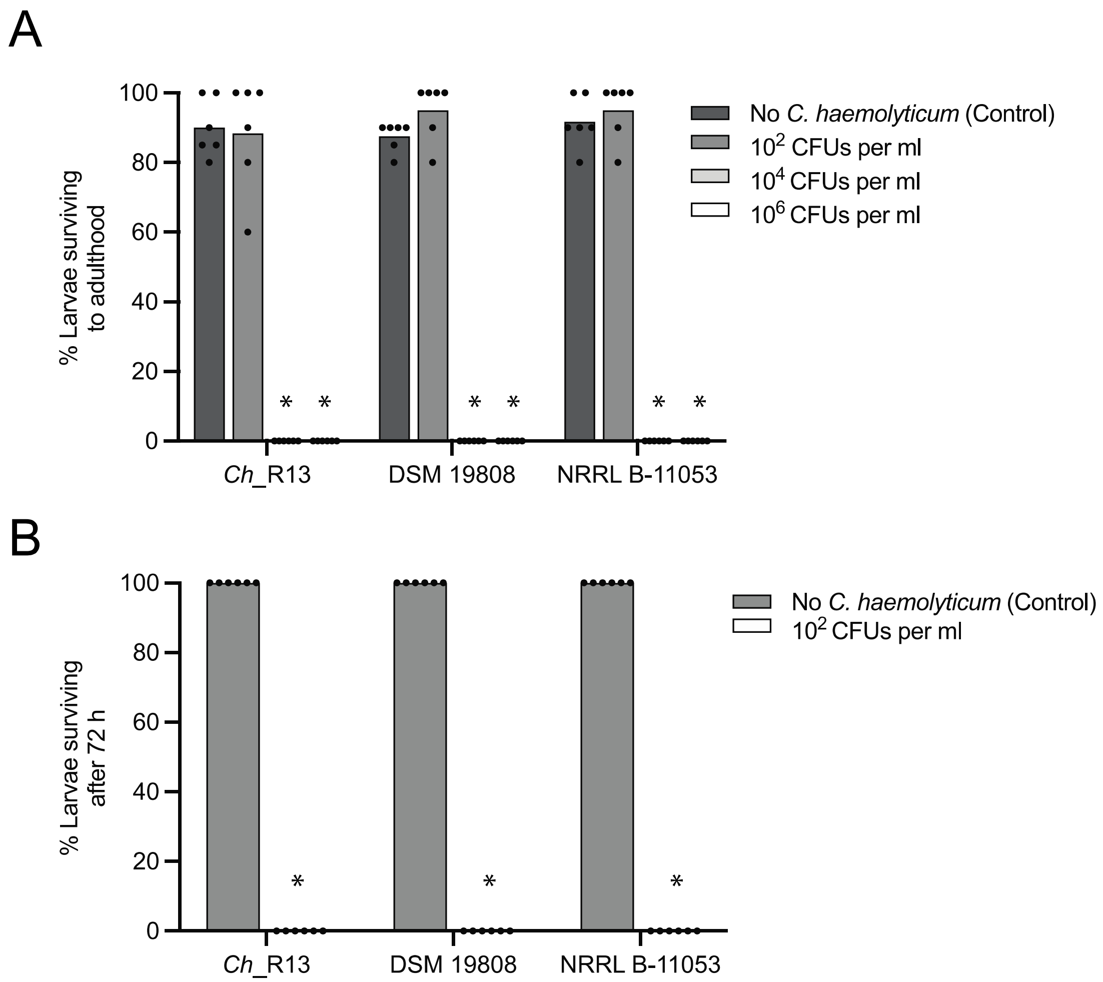

Fig S6 *Ch*_R13, DSM 19808 and NRRL B-11053 have similar mortality effects in CN than AX cultures of *A. aegypti* larvae. (A) Percentage of first instar *A. aegypti* in CN cultures that develop into adults when no *C. haemolyticum* is added to a culture well (Control) versus when *Ch*_R13, DSM 19808 or NRRL B-11053 is added at a starting density of 1 x 10^2, 10^4 or 10^6 CFUs per ml. Each replicate is a culture well with 10 larvae (6 replicates per treatment.) Columns show mean values for each treatment with each replicate shown as solid circles. For each instar, an asterisk (*) indicates a significant difference for a given treatment when compared to the Control as determined by a Fisher's exact test ($P<0.0001$). (B) Percentage of first instar *A. aegypti* in AX cultures that are alive after 72 h when no *C. haemolyticum* strain is added to culture wells (Control) versus when *Ch*_R13, DSM 19808 or NRRL B-11053 is added at a starting density of 10^2. Columns, solid dots, and asterisks are defined as in (A).

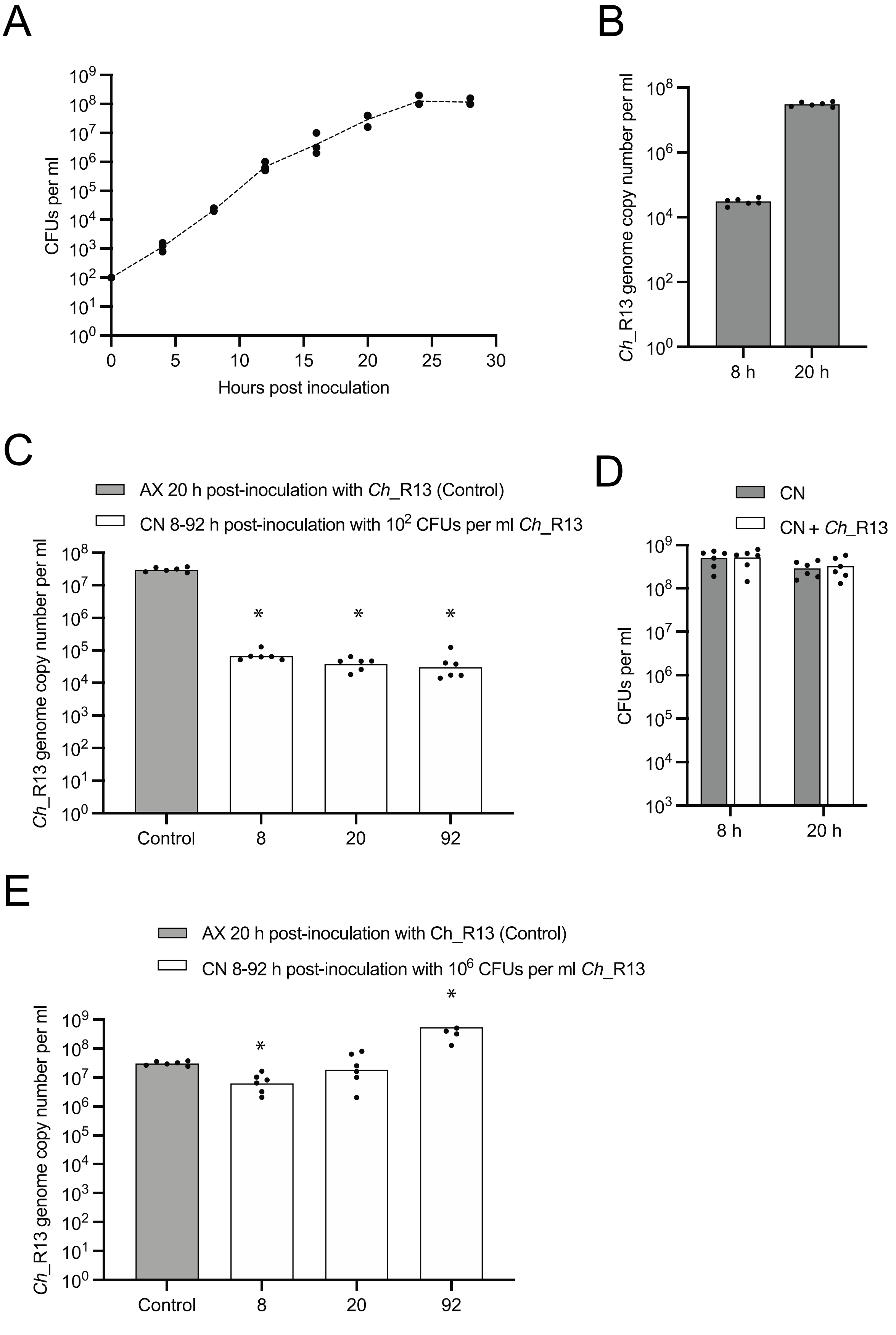
A
CFUs per ml
Hours post inoculation
B
Ch_R13 genome copy number per ml
8 h
20 h
C
AX 20 h post-inoculation with Ch_R13 (Control)
CN 8-92 h post-inoculation with 10^2 CFUs per ml Ch_R13
Ch_R13 genome copy number per ml
Control
8
20
92
D
CN
CN + Ch_R13
CFUs per ml
8 h
20 h
E
AX 20 h post-inoculation with Ch_R13 (Control)
CN 8-92 h post-inoculation with 10^6 CFUs per ml Ch_R13
Ch_R13 genome copy number per ml
Control
8
20
92

Fig S7 *Ch*_R13 grows to higher densities in AX than CN cultures. (A) Growth of *Ch*_R13 in AX cultures from a starting density of 10^2 CFUs (two replicates) as measured by colony counts. (B) *Ch*_R13 abundance in AX cultures at 8 and 20 h post-inoculation as estimated by a qPCR assay that measured copy number of the single copy *gyrA* gene. Starting density was 10^2 CFUs per ml. Columns show mean genome copy number per ml while solid circles show copy number per replicate which was a culture well containing 5 ml of sterile water and sterile larval RCM rearing diet (6 independent replicates per treatment). (C) Genome copy of *Ch*_R13 in an AX culture at 20 h (Control) versus genome copy number in a CN culture from 8-92 h of adding *Ch*_R13 at 10^2 CFUs per ml. Columns and solid circles are as defined in (B). Asterisks (*) indicate CN treatments that significantly differ from the AX 20 h control ($F_{3,20}$ = 253.4, P <0.0001 followed by a post-hoc Dunnett's test a=0.05). (D) Abundance of bacteria culturable on LB plates in CN cultures versus CN cultures to which *Ch*_R13 was added at a starting density of 10^2 CFUs per ml at 8 and 20 h post-inoculation. Columns and solid circles are as defined in (B). t-tests detected no differences between treatments (P>0.05). (E) Genome copy of *Ch*_R13 in an AX culture at 20 h (Control) versus genome copy number in CN cultures from 8-92 h of adding *Ch*_R13 at 10^6 CFUs per ml. Columns and solid circles are as defined in (B). Asterisks (*) indicate CN treatments that significantly differed from the AX 20 h control ($F_{3,20}$ = 25.1, P <0.0001 followed by a post-hoc Dunnett's test a=0.05). The AX data sets used in (C) and (E) are the same as the data set at 20 h used in (B).

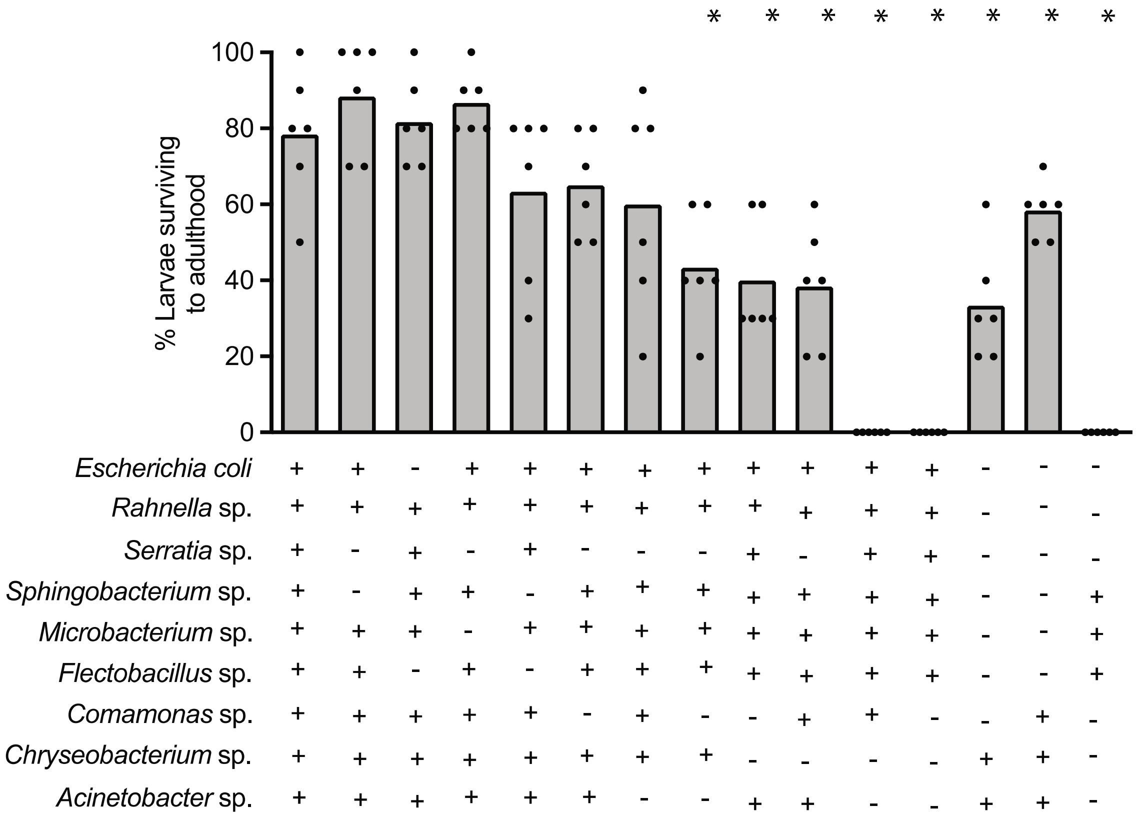

Fig S8 Percentage of first instar *A. aegypti* that develop into adults in GN cultures with the ALL9 community, or GN cultures with 2-7 members of the ALL9 community present when *Ch*_R13 is added at a starting density of 1 x 10^2 CFUs per ml. Columns, solid circles and asterisks as defined in (A). The same ALL9 data set is used in each graph.

A

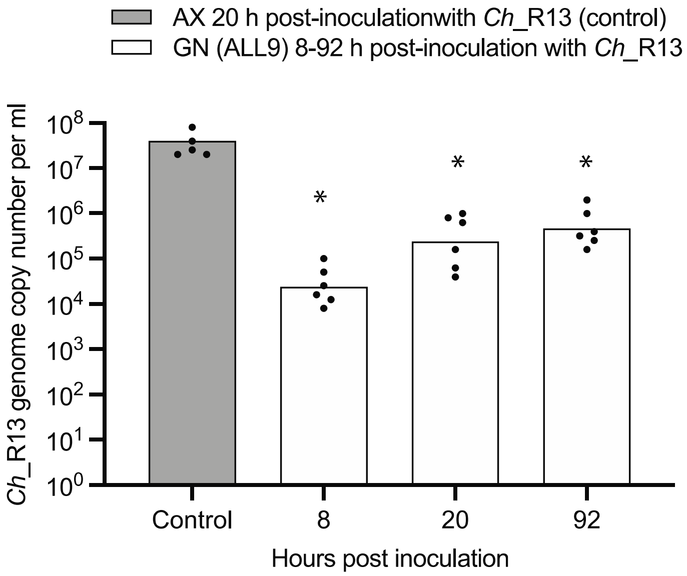

B

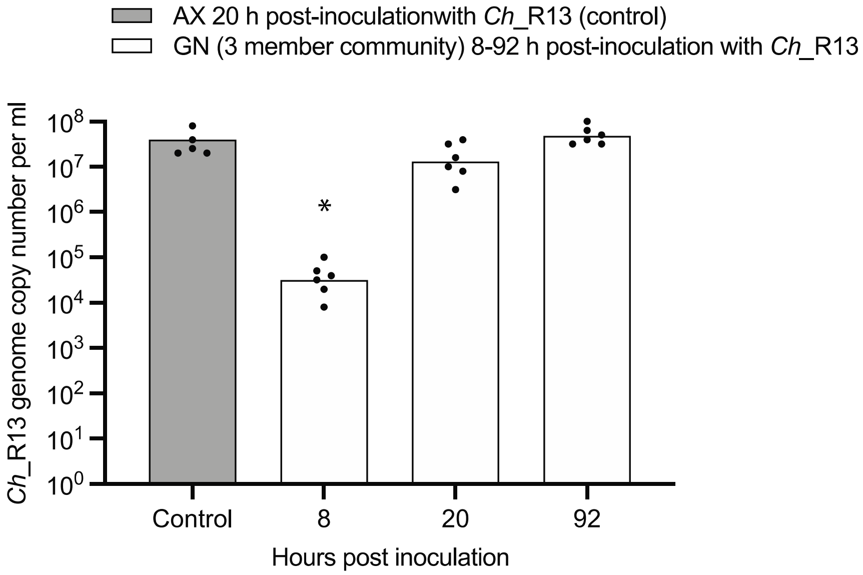

Fig S9 Starting density affects the abundance of of *Ch*_R13 in GN cultures containing the ALL9 community when compared to AX cultures with no microbiota. (A) Genome copy of *Ch*_R13 in an AX culture at 20 h (Control) versus genome copy number in GN cultures with the ALL9 community; 8-92 h of adding *Ch*_R13 at 10^2 CFUs per ml. Columns and solid circles as defined in Fig. S6C. Asterisks (*) indicate abundance of *Ch*_R13 in the GN treatments remained lower

than in the AX 20 h control ($F_{3,20}$ = 53.9, P <0.0001, P <0.0001 followed by a post-hoc Dunnett's test a=0.05). (B) Genome copy of *Ch*_R13 in an AX culture at 20 h (Control) versus genome copy number in GN cultures containing *Spingobacterium* sp., *Microbacterium* sp. and *Flectobacillus* sp. 8-92 h after adding *Ch*_R13 at 10^2 CFUs per ml. Columns and solid circles in (A) and (B) as defined in Fig. S6C. Asterisks (*) indicate abundance of *Ch*_R13 in the GN treatments remained lower than in the AX 20 h control ($F_{3,20}$ = 119.3, P <0.0001, P <0.0001 followed by a post-hoc Dunnett's test a=0.05). Columns and solid circles are as defined in (B). Asterisks (*) indicate abundance of *Ch*_R13 in the GN treatments rose to control levels ($F_{5,30}$ = 38.7, P <0.0001 followed by a post-hoc Dunnett's test a=0.05).

A

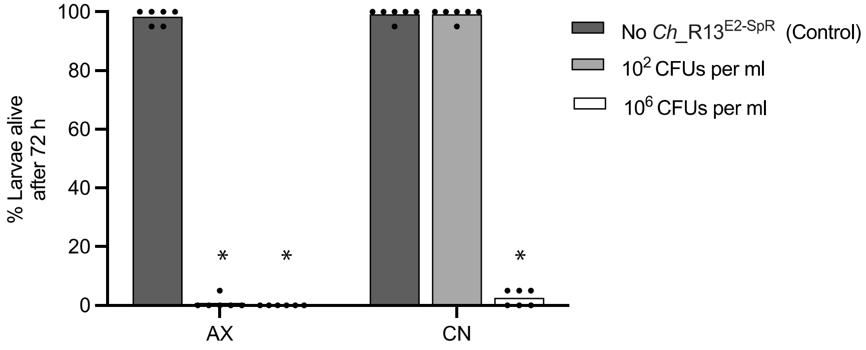

100
80
60
40
20
0
% Larvae alive after 72 h
*
*
*
AX
CN
No Ch_R13E2-SpR (Control)
10^2 CFUs per ml
10^6 CFUs per ml

B

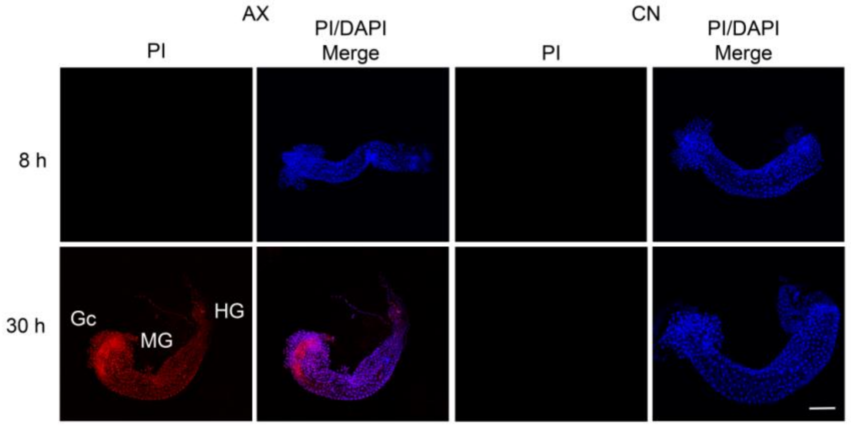

AX
CN
PI
PI/DAPI Merge
PI
PI/DAPI Merge
8 h
30 h
Gc
MG
HG

Fig S10 *Ch*_R13$^{E2\text{-}SpR}$ kills *A. aegypti* larvae. (A) Percentage of first instars in AX and CN cultures that are alive after 72 h when no *Ch*_R13$^{E2\text{-}SpR}$ are added to culture wells (Control) versus when *Ch*_R13$^{E2\text{-}SpR}$ is added at a starting of 10^2 or 10^6 CFUs per ml. Columns, solid dots, and asterisks are defined as in Fig. S7. (B). Confocal microscopy images showing the midgut and hindgut from first instars 8 and 30 h after infection of AX or CN cultures with *Ch*_R13 at a starting density of 1 x 10^2 CFUs per ml. Samples were stained with propidium iodide (PI) and DAPI. The digestive tract in each panel is oriented with the gastric caeca (Gc) and anterior midgut oriented to the left and the hindgut (HG) to the right. Scale bar = 50 um.

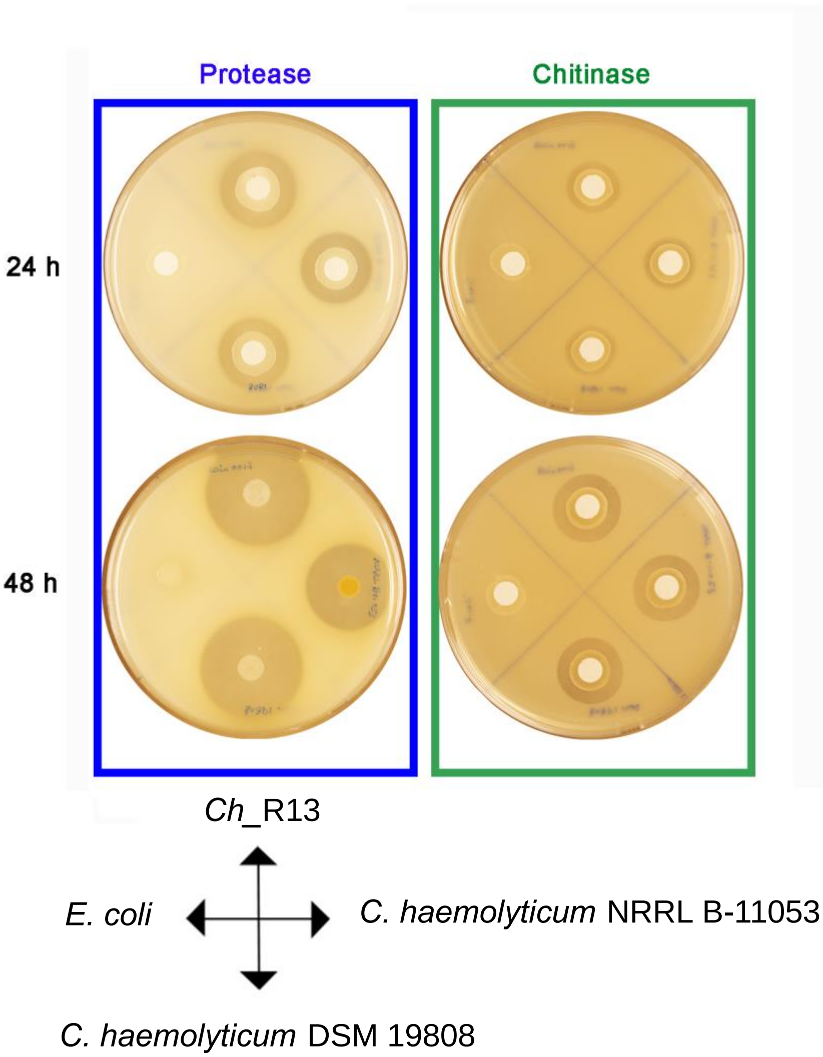

Fig S11 Plate assays showing that *Ch*_R13, *C. haemolyticum* DSM19808 and *C. haemolyticum* NRRL B-11053 exhibit protease by clearance on milk agar plates and chitinase activity by clearance on chitin agar plates. *E. coli* K12 (negative control) exhibited no protease or chitinase activity.

SI Materials and Methods

Mosquitoes

The University of Georgia (UGAL) strain of *Aedes aegypti* was established from wild-caught females in Athens, GA (2). The MRA-804 strain of *Aedes albopictus* was obtained from the Centers for Disease Control and Prevention (CDC) in Atlanta, GA, USA and has been in our insectary since 2012. The CDC MR4/BEI strain of *Culex quinquefasciatus* has been in our insectary since 2011. All mosquitoes were reared under a 12 h light:12 h dark photoperiod at 26°C and 70% relative humidity. Larvae of each species hatch from eggs and grow through four instars before pupating. Each was reared in pans at a density of ~150 larvae per liter of deionized water and fed daily rearing diet until pupation at ~6 days post-hatch. Rearing diet consisted of ground rat chow pellets (LabDiet 5001), lactalbumin (Sigma), and torula yeast extract (Bio-Serve) mixed in a ratio of 1:1:1 by volume, referred to as rat chow mix (RCM) (3). Adult mosquitoes were provided water and 10% sucrose in water *ad libitum*. For colony maintenance, adults from each generation were blood-fed 3-5 days post-eclosion to obtain eggs. Adults were blood-fed from an anesthetized male lab rat (Sprague Dawley), while *An. gambiae* was fed defibrinated rabbit blood (Hemostat Laboratories) using a membrane feeder. Anesthetization of rats and feeding were monitored by trained personnel following guidelines from the approved animal use protocol A2020 12-008-R1 approved by the University of Georgia Institutional Animal Care and Use Committee (IACUC), which is licensed by the US Department of Agriculture (USDA) and maintains an animal welfare Assurance, in compliance with Public Health Service policy, through the NIH Office of Laboratory Animal Welfare, and registration with the USDA APHIS Animal Care, in compliance with applicable federal regulations, guidance, and state laws governing animal use in research and teaching. Blood fed females were provided cups lined with wetted paper towel to oviposit upon 2-4 days post-blood feeding.

Bacteria

Bacteria used in the study and their origins are listed in Table S1. In addition to *Ch*_R13, these included *Chromobacterium haemolyticum* DSM 19808; *C. haemolyticum* NRRL B-11053, and eight commensal species (*Microbacterium* sp., *Sphingobacterium* sp., *Flectobacillus* sp., *Rahnella* sp., *Serratia* sp., *Acinetobacter* sp., *Chryseobacterium* sp., *Comamonas* sp.) we earlier isolated from CN laboratory or field cultures (4, 5). We also used *Escherichia coli* K-12 substrain MG1655. *E. coli* has been identified in other *A. aegypti* cultures (5), while we have previously used this K12 MG1655 to produce monoxenic, GN *A. aegypti* cultures that develop into adults (6). Each of the above bacteria were stored as glycerol stocks at -80° C. Together, the eight commensal species plus *E. coli* K12 comprised the ALL-9 community described below that was used to produce gnotobiotic (GN) mosquito cultures.

Isolation, whole genome sequencing, and phylogenetic analysis of *Ch*_R13

Water from an outdoor container in Athens, GA USA named "Rain" was collected in September 2017 and returned to the laboratory where particulate detritus was removed by low-speed centrifugation at 250 x *g* followed by centrifugation of the supernatant at 6000 x *g* for 15 min. The resulting pellet was suspended in a 1:1 mixture of sterile glycerol:1x PBS and cryopreserved at -80°C. Bacteria in the glycerol stock were plated on 1/10 diluted 869 agar plates and cultured at 25° C. Unique colony morphologies were selected and passaged three times to new agar plates to ensure individual isolates. Isolates were then suspended in a 1:1 mixture of sterile glycerol:1x PBS and cryopreserved at -80°C. The DNeasy Blood and Tissue kit (Qiagen, Valencia, CA, USA) was used to isolate DNA from colonies exhibiting larvicidal activity (see below). DNA templates from these colonies were used to amplify a portion of the 16S rRNA gene with the primer set 27F short (5'-AGAGAGTTTGATCCTGGCTCAG-3') 1507R (5'-TACCTTGTTACGACTTCACCCCAG-3'), HotMaster Taq DNA polymerase (Quantabio, Beverly, MA, USA), and previously described polymerase chain reaction (PCR) conditions (3, 7, 8). Amplicons were visualized on a 1%

agarose gel and cleaned with the QIAquick PCR purification kit (Qiagen, Valencia, CA, USA) before submitting for Sanger sequencing at Eurofins Genomics (Louisville, KY, USA). Resulting sequences were then compared to the NCBI nr database using BLASTn.

The isolate from the "Rain" container identified as colony Rain0013 was further studied by extracting DNA from cells grown on 1/10 diluted 869 agar at 25° C using phenol/chloroform (Pacific Biosciences, https://www.pacb.com/wp-content/uploads/2015/09/SharedProtocol-Extracting-DNA-usinig-Phenol-Chloroform.pdf). After quantification and quality checks (gel electrophoresis, nanodrop), DNA was sequenced using PacBio Sequel system at the UGA Georgia Genomics and Bioinformatics Core. A circular chromosome for this bacterium, designated as *Ch*_R13 (see Results), was assembled from reads with Canu version 1.7 (9) and polished with arrow in smrtlink version 5.1 (Pacific Biosciences, Menlo Park, CA, USA). The circular chromosome was further assessed with Circlator (10). The NCBI Prokaryotic Annotation pipeline (PGAP) was used to assign gene identifications (11). Prophage elements were predicted with PHASTER (12).

Different *Chromobacterium* genomes were compared to Ch_R13, following contig alignment and rearrangement with progressiveMauve (13) implemented in Geneious (Geneious 2022.2.1). A circular representation of the genome was generated with Proksee accessed in June 2022 (14), which is an updated version of the CGView Server (15). Multi-contig genome assemblies were merged using merge-gbk-records command "merge-gbk-records -s stop $infile.gbff > $outfile.gbff" (https://github.com/kblin/merge-gbk-records). Genomic synteny and genomic island content were assessed among *Chromobacterium* spp. using Island Compare (16) and virulence-related genes were identified using the Virulence Factor Database (VFDB) (17). To identify secretion systems, proteins for each genome was functionally annotated with the HMM-based KEGG ortholog assignment tool KofamKOALA (18) and mapped to metabolic pathways with KEGG Mapper (19). Type VI secretion system (T6SS) operons were compared among select strains of *C. haemolyticum* and *C. substugae* using clinker (20).

A genome-level phylogeny was built with all *Chromobacterium* genome assemblies in Genbank (accessed June 2022) and several closely related Betaproteobacteria for a total of 117 taxa (accession numbers listed in the phylogeny) using GToTree v1.6.31 under default parameters, described here briefly (21). The set of 203 “Betaproteobacteria” single-copy orthologous genes was extracted from each genome, aligned, translated into amino acids, trimmed, and concatenated with several programs: Prodigal v2.6.3 (22), HMMER3 v3.3.2 (23), Muscle v5.1 (https://www.drive5.com/muscle/), TrimAl v1.4.rev15 (24), TaxonKit v0.9.0 (25). With this input, FastTree 2 v2.1.11 (26) was used to infer an approximate maximum likelihood phylogenetic tree, with a JTT + CAT amino acid substitution model. Local support values were calculated with 1000 Shimodaira–Hasegawa (SH)-like replicates. Species and strain information were added using the parameters -t -L Species,Strain. For the *C. haemolyticum* group phylogeny, outputs of FastTree2 (amino acid alignment and Partitions.txt) were used by the IQ-TREE 1.6.12 webserver with 1000 ultrafast bootstrapping replicates for support (27-29). ModelFinder (30) was used to select a model for each partition (25 taxa, 201 partitions, 44918 total sites, 3.46 % missing data). The resulting maximum likelihood tree with bootstrap support was visualized using the Interactive Tree of Life v6.5.7 (iTOL) web interface (31).

Transformation

The plasmid pBTK570 encoding the E2-Crimson gene and resistance to spectinomycin was transformed into the donor strain of *E. coli* MFD*pir* (Mu-free donor) which is a diaminopimelic acid (DAP) auxotroph (32) by electroporation (32). pBTK570 was transferred to *Ch*_R13 through conjugation by first growing the recipient in Luria broth (LB) and the donor in LB plus antibiotic. After pelleting and resuspending each in sterile Tris buffer, 100 µl of the recipient strain was spread on an LB plus spectinomycin (50 mg/ml) plate to dryness followed by addition of 100 µl of the donor. Transconjugates were then selected for by overnight incubation at 30° C. Resulting strains were grown to midlog phase and then pelleted and resuspended in water for use in

bioassays. Visibly fluorescent colonies were passaged to new selective media, and the 16S rRNA gene was sequenced (Eurofins Genomics, Louisville, KY, United States) to ensure correct species identity. Gycerol stocks were prepared to cryopreserve at -80°C the strain the strain used in assays (*Ch*_R13$^{E2\text{-}SpR}$).

Axenic (AX), gnotbiotic (GN) and conventional (CN) mosquito cultures

AX larvae were produced by surface sterilizing *A. aegypti* eggs as previously described with resulting first instars hatching in sterile water (6). Ten first instars were then transferred to 6-well plates containing 5 ml of sterile water plus RCM diet per well to produce AX cultures whose sterility was confirmed using previously described PCR and culture-based assays (3, 6). AX second-fourth instars used in assays were produced in darkness under AX rearing conditions as previously described using LP:YE diet (34) followed by transfer of larvae to culture wells containing 5 ml of sterile water plus RCM diet as used above for first instars. GN cultures with a defined community of commensal microbes were produced by adding one or members of the ALL9 community to AX cultures, which were grown and added as previously detailed (3). CN cultures were produced by placing first-fourth instar larvae from our general culture into 6-well culture plates containing 5 ml of sterile water plus RCM diet per well.

Larvicidal assays

The first larvicidal assays we conducted used 20 ml of water from container "Rain" (see above), sterile RCM diet and 20 CN *A. aegypti, A. albopictus* or *C. quinquefasciatus* first instars were added to 25 cm culture flasks (Genesee). Larvae were then observed every 24 h to assess the number of alive versus dead individuals present. The same assays were also conducted using sterile water or "Rain" water that was autoclaved or filter sterilized through an 0.2 µm filter (Millipore), which was then held at 4° C for many days before being retested. Assays using different strains of *C. haemolyticum* were conducted by growing bacteria in Luria Broth (LB) or

sterile water containing sterile larva RCM rearing diet with abundance monitored by optical density (OD) at 600 nm while density was determined by concurrently collecting samples that were serially diluted to determine colony forming units (CFUs) per ml as previously described (3, 35). Bacteria were added at a starting density of $1x10^2$-$1x10^6$ CFUs per ml to CN, GN or AX cultures in 6 well culture plates (5 ml per well) containing 10 *A. aegypti* larvae (first-fourth instars) which were then examined at specific times post-inoculation to determine the number of alive versus dead larvae present. The unit of replication for all of the larvicidal assays was thus a culture well which contained a starting density of 10 or 20 CN, GN or AX larvae. Each treatment for a given assay was replicated a minimum of 6 times using culture wells that were established from independently generated starting pools of larvae and microbes. The percentage of surviving larvae or larvae that developed into adults per replicate were then compared to a control treatment that also consisted of 6 independently generated replicates. Data analysis is described in the Methods reported in the main text.

*Ch*_R13 densities in AX cultures were determined using colony counts as described above, while densities in CN cultures were estimated using a quantitative PCR (qPCR) assay that used specific primers (forward 5'-ACAAGATCGTCGCCGAATAC-3', reverse 5'-TAACAGCGGACAACTCATCG-3") to measure abundance of the single copy *gyrase A* gene. Specificity of this amplicon was confirmed by PCR using DNA from *Ch*_R13 or bacteria from CN cultures where no product was detected. Bacterial DNA in CN cultures was isolated using the Qiagen DNeasy Blood and Tissue kit. After extraction, qPCR assays were run on a Rotor Gene Q (Qiagen): 3 min initial denaturation step at 95 °C, followed by 40 cycles of 20 s, denaturation at 95 °C, 20 s annealing at 50 °C and 20 s, extension at 72 °C. Data were acquired during the extension step and analyzed using Rotor-Gene application software. All reactions were conducted in quadruplicate. The data were then fit to a standard curve constructed using known amounts of the plasmid pTOPO TA vector containing the *gyrase* A amplicon that was serially diluted (36) to estimate genome copy number per ml of water. As with the larvicidal assays, the

unit of replication for measuring *Ch*_R13 densities was a culture well while CFUs/ml or genome copies/ml for each treatment in a given assay was replicated a minimum of 6 times using independently generated samples. Data analysis is described in the Methods reported in the main text.

Cyanide concentrations and associated assays

*Ch*_R13 at a starting density of 10^2 CFUs per mL was inoculated into culture wells containing 5 ml of sterile water and sterile mosquito RCM diet as used in larvicidal assays. Growth of bacteria was then monitored by measuring optical density at 600 nm using a plater reader (Biotek Synergy) and colony forming units per ml as described above using water samples collected at different times. Cyanide concentrations were measured by an established assay (37). Briefly, small aliquots of water were collected from the above cultures at the same times that bacterial abundances were measured. A 100 mM dinitrobenzene (Sigma) and a 200 mM p-nitrobenzaldehyde (Sigma) solution were prepared in 2-methoxyethanol (Sigma). A fresh 1:1 mixture of these two solutions was mixed with the experimental sample (77:23) to 100 µl followed by addition of 1.8 µl of NaOH. After 30 min at room temperature, 900 µl of 2-methoxyethanol was added followed measuring aliquots of each sample at 578 nm using the above plate reader with cyanide concentration determined by fitting the data to a standard curve generated by serial dilution of a potassium cyanide (KCN) stock solution. These assays which generated growth curves for *Ch*_R13 and associated measures of cyanide concentration in the water were measured three times using independently prepared starting samples. Effects on *A. aegypti* larvae were assessed using water containing cyanide from *Ch*_R13 cultures that was sterilized through a 0.2 µm filter (Pall) and serially diluted. One ml volumes of this water or a serially diluted KCN solution was placed in open 24 well culture plates (Genesee) followed by addition of 10 first instar *A. aegypti* and a small amount of sterile RCM rearing diet followed by assessment of the number of alive and dead larvae at different times. A hydroxocobalamin (vitamin B12a) (TCI

America) stock solution was prepared in sterile water with 10 mM added to water containing a starting cyanide concentration of 10 mM immediately before addition of larvae or larvae and $1x10^2$ CFUs of *Ch*_R13. The number of living and dead larvae as were assessed 1 and 24 h later. Larvae were scored as living if mobile while larvae were scored as dead if immobile. Dead larvae also rapidly become more opaque due discoloration of the hemocoel. For these assays, culture wells containing a starting density of 10 larvae were the unit of replication. Each treatment was replicated a minimum of 6 times using independently prepared samples as described above for the larvicidal assays. Data analysis is described in the Methods reported in the main text.

Immunofluorescence microscopy

AX cultures containing *A. aegypti* first instars were inoculated with 1×10^2 CFUs per ml of *Ch*_R13$^{E2\text{-}SpR}$ followed by collection of larvae at different times and dissection of the digestive tract in in phosphate buffer saline (PBS, pH 7.4). Samples were fixed in 4% paraformaldehyde in PBS for 20 min at room temperature. After rinsing three times in PBS, guts were permeabilized for 20 min in PBS plus 0.2% Triton X-100 (PBT) for 20 min and incubated with F432 Fluorescein Phalloidin (Thermo Fisher) at room temperature for 1 h. After rinsing, samples were slide mounted in glycerol containing HOECHST 33342 (1 µg per ml). For vital dye staining, AX and CN cultures containing first instars were inoculated with *Ch*_R13 followed by collection of digestive tracts that were incubated with propidium iodide (Thermo Fisher) at room temperature for 20 min followed by addition of HOECHST 33342 (1 µg per ml). All samples were then slide-mounted with cover slips and examined using a Zeiss LSM 710 inverted confocal microscope with acquired images processed using Adobe Photoshop.

Protease and chitinase assays

Milk agar plates were used to assess protease activity and chitin agar plates were used to measure chitinase activity (38). Milk agar plates were made by adding 2.5 g yeast extract and 7.5

g agar to 350 ml of water; 15 g of milk powder was dissolved in 150 ml of water; both solutions were then autoclaved, cooled, mixed, and poured into plates. Blood agar plates were made using LB and sterile rabbit blood. Chitin powder (20 g) was dissolved in 500 ml of concentrated hydrochloric acid and continuously stirred at 4°C for 1 h. The hydrolyzed chitin was washed several times with distilled water to remove the acid and to bring the pH to the range of 6 to 7. The colloidal chitin was then filtered and stored at 4°C. Chitin plates were made as follows: 7 g of K_2HPO_4, 0.5 g of $MgSO_4$ heptahydrate, 2 g of yeast extract, and 15 g of agar were dissolved in 850 ml of water; 20 g of colloidal chitin was dissolved in 150 ml of water; both solutions were then autoclaved, cooled, mixed, and poured into plates. Each plate was quartered followed by addition of a 7 mm circular piece of sterile filter paper to which 10^9 CFUs of *Ch*_R13, *C. haemolyticum* NRRL B-11053, *C. haemolyticum* DSM 19808 or *E. coli* K12 was spotted. Plates were incubated at 30° C with enzymatic activity qualitatively assessed 24 and 48 h later.

REFERENCES

1. Shyntum DY, Venter SN, Moleleki LN, Toth I, Coutinho TA. 2014. Comparative genomics of type VI secretion systems in strains of *Pantoea ananatis* from different environments. BMC Genomics 15:163. https://doi.org/10.1186/1471-2164-15-163
2. Foster WA, Lea AO. 1975. Renewable fecundity of male *Aedes aegypti* following replenishment of seminal vesicles and accessory glands. J Insect Physiol 21:1085–1090. https://doi.org/10.1016/0022-1910(75)90120-1
3. Martinson VG, Strand MR. 2021. Diet–microbiota interactions alter mosquito development. Front Microbiol 12:650743. https://doi.org/10.3389/fmicb.2021.650743
4. Coon KL, Brown MR, Strand MR. 2016. Mosquitoes host communities of bacteria that are essential for development but vary greatly between local habitats. Mol Ecol 25:5806–5826. https://doi.org/10.1111/mec.13877

5. Thongsripong P, Chandler J.A, Green AB, Kittayapong P, Wilcox BA, Kapan DD, Bennett SN. 2018. Mosquito vector-associated microbiota: Metabarcoding bacteria and eukaryotic symbionts across habitat types in Thailand endemic for dengue and other arthropod-borne diseases. Ecol Evol 8:1352–1368. https://doi.org/10.1002/ece3.3676
6. Coon KL, Vogel KJ, Brown MR, Strand MR. 2014. Mosquitoes rely on their gut microbiota for development. Mol Ecol 23:2727–2739. https://doi.org/10.1111/mec.12771
7. Coon KL, Valzania L, McKinney DA, Vogel KJ, Brown MR, Strand MR. 2017. Bacteria-mediated hypoxia functions as a signal for mosquito development. Proc Natl Acad Sci USA 114:E5362-E5369. https://doi.org/10.1073/pnas.1702983114
8. Vogel KJ, Valzania L, Coon KL, Brown MR, Strand MR. 2017. Transcriptome sequencing reveals large-scale changes in axenic Aedes aegypti larvae. PLoS Negl Trop Dis 11: e0005273. https://doi.org/10.1371/journal.pntd.000527
9. Koren S, Walenz BP, Berlin K, Miller JR, Bergman NH, Phillippy AM. 2017. Canu: Scalable and accurate long-read assembly via adaptive k -mer weighting and repeat separation. Genome Res 27:722–736. https://doi.org/10.1101/gr.215087.116
10. Hunt M, Silva ND, Otto TD, Parkhill J, Keane JA, Harris SR. 2015. Circlator: Automated circularization of genome assemblies using long sequencing reads. Genome Biol 16:294. https://doi.org/10.1186/s13059-015-0849-0
11. Tatusova T, DiCuccio M, Badretdin A, Chetvernin V, Nawrocki EP, Zaslavsky L, Lomsadze A, Pruitt KD, Borodovsky M, Ostell J. 2016. NCBI prokaryotic genome annotation pipeline. Nuc Acids Res 44:6614–6624. https://doi.org/10.1093/nar/gkw569
12. Arndt D, Grant JR, Marcu A, Sajed T, Pon A, Liang Y, Wishart DS. 2016. PHASTER: A better, faster version of the PHAST phage search tool. Nuc Acids Res 44(W1):W16–W21. https://doi.org/10.1093/nar/gkw387

13. Darling AE, Mau B, Perna NT. 2010. progressiveMauve: Multiple genome alignment with gene gain, loss and rearrangement. PLoS ONE, 5:e11147. https://doi.org/10.1371/journal.pone.0011147

14. Grant JR, Enns E, Marinier E, Mandal A, Herman EK, Chen C, Graham M, Van Domselaa G, Stothard P. 2023. Proksee: In-depth characterization and visualization of bacterial genomes. Nuc Acids Res 51:W484-W492. https://doi.org/10.1093/nar/gkad326

15. Grant JR, Stothard P. 2008. The CGView server: A comparative genomics tool for circular genomes. Nuc Acids Res 36:W181–W184. https://doi.org/10.1093/nar/gkn179

16. Bertelli C, Gray KL, Woods N, Lim AC, Tilley KE, Winsor GL, Hoad GR, Roudgar A, Spence, A, Peltier J, Warren D, Raphenya AR, McArthur AG, Brinkman FSL. 2022. Enabling genomic island prediction and comparison in multiple genomes to investigate bacterial evolution and outbreaks. Microb Genom 8:mgen000818. https://doi.org/10.1099/mgen.0.000818

17. Liu B, Zheng D, Zhou S, Chen L, Yang J. 2022. VFDB 2022: A general classification scheme for bacterial virulence factors. Nuc Acids Res 50:D912–D917. https://doi.org/10.1093/nar/gkab1107

18. Aramaki T, Blanc-Mathieu R, Endo H, Ohkubo K, Kanehisa M, Goto S, Ogata H. 2020. KofamKOALA: KEGG Ortholog assignment based on profile HMM and adaptive score threshold. Bioinformatics 36:2251–2252. https://doi.org/10.1093/bioinformatics/btz859

19. Kanehisa M, Sato Y. 2020. KEGG Mapper for inferring cellular functions from protein sequences. Protein Sci 29:28–35. https://doi.org/10.1002/pro.3711

20. Gilchrist CL M, Chooi Y-H. 2021. Clinker & clustermap.js: Automatic generation of gene cluster comparison figures. Bioinformatics, 37:2473–2475. https://doi.org/10.1093/bioinformatics/btab007

21. Lee MD. 2019. GToTree: A user-friendly workflow for phylogenomics. Bioinformatics, 35:4162–4164. https://doi.org/10.1093/bioinformatics/btz188

22. Blom D, Fabbri C, Eberl L, Weisskopf L. 2011. Volatile-mediated killing of *Arabidopsis thaliana* by bacteria Is mainly due to hydrogen cyanide. Appl Env Microbiol 77:1000–1008. https://doi.org/10.1128/AEM.01968-10
23. Finn RD, Clements J, Eddy SR. 2011. HMMER web server: Interactive sequence similarity searching. Nuc Acids Res 39(suppl):W29–W37. https://doi.org/10.1093/nar/gkr367
24. Capella-Gutiérrez S, Silla-Martínez JM, Gabaldón T. 2009. trimAl: A tool for automated alignment trimming in large-scale phylogenetic analyses. Bioinformatics, 25:1972–1973. https://doi.org/10.1093/bioinformatics/btp348
25. Shen W, Ren H. 2021. TaxonKit: A practical and efficient NCBI taxonomy toolkit. J Genet Genomics, 48:844–850. https://doi.org/10.1016/j.jgg.2021.03.006
26. Price MN, Dehal PS, Arkin AP. 2010. FastTree 2 – Approximately maximum-likelihood trees for large alignments. PLoS ONE, 5:e9490. https://doi.org/10.1371/journal.pone.0009490
27. Nguyen L-T, Schmidt HA, von Haeseler A, Minh BQ. 2015. IQ-TREE: A fast and effective stochastic algorithm for estimating maximum likelihood phylogenies. Mol Biol Evol 32:268-274. https://doi.org/10.1093/molbev/msu300
28. Hoang DT, Chernomor O, von Haeseler A, Minh BQ, Vinh LS. 2018 UFBoot2: Improving the ultrafast bootstrap approximation. Mol Biol Evol 35:518-522. https://doi.org/10.1093/molbev/msx281
29. Chernomor O, von Haeseler A, Minh BQ. 2016 Terrace aware data structure for phylogenomic inference from supermatrices. Syst Biol 65:997-1008. https://doi.org/10.1093/sysbio/syw037
30. Kalyaanamoorthy S, Bui Quang Minh BQ, Wong TKF, von Haeseler A, Jermiin LS. 2017 ModelFinder: Fast model selection for accurate phylogenetic estimates. Nat Methods 14, 587–589. https://doi.org/10.1038/nmeth.4285

31. Letunic I, Bork P. 2016. Interactive tree of life (iTOL) v3: An online tool for the display and annotation of phylogenetic and other trees. Nuc Acids Res 44:W242–W245. https://doi.org/10.1093/nar/gkw290

32. Ferrières L, Hémery G, Nham T, Guérout A-M, Mazel D, Beloin C, Ghigo J-M. 2010. Silent mischief: Bacteriophage Mu insertions contaminate products of *Escherichia coli* random mutagenesis performed using suicidal transposon delivery plasmids mobilized by broad-host-range RP4 conjugative machinery. J Bact 192:6418–6427. https://doi.org/10.1128/JB.00621-10

33. Leonard SP, Perutka J, Powell JE, Geng P, Richhart DD, Byrom M, Kar S, Davies BW, Ellington AD, Moran NA, Barrick JE. 2018. Genetic engineering of bee gut microbiome bacteria with a toolkit for modular assembly of broad-host-range plasmids. ACS Synthetic Biol 7:1279–1290. https://doi.org/10.1021/acssynbio.7b00399

34. Wang Y, Eum JH, Harrison RE, Valzania L, Yang X, Johnson JA, Huck DT, Brown MR, Strand MR. 2021. Riboflavin instability is a key factor underlying the requirement of a gut microbiota for mosquito development. Proc Natl Acad Sci 118:e2101080118. https://doi.org/10.1073/pnas.2101080118

35. Thomas P, Sekhar AC, Upreti R, Mujawar MM, Pasha SS. 2015 Optimization of single plate-serial dilution spotting (SP-SDS) with sample anchoring as an assured method for bacterial and yeast cfu enumeration and single colony isolation from diverse samples. Biotechnol Rep 8:45-55. https://doi.org/10.1016/j.btre.2015.08.003

36. Beck MH, Inman RB, Strand MR. 2007. Microplitis demolitor bracovirus genome segments vary in abundance and are individually packaged in virions. Virology, 359:179–189. https://doi.org/10.1016/j.virol.2006.09.002

37. Guilbault GG, Kramer DN. 1966. Ultra sensitive, specific method for cyanide using p -nitrobenzaldehyde and o -dinitrobenzene. Anal Chem 38:834–836. https://doi.org/10.1021/ac60239a009

38. Raymann K, Coon KL, Shaffer Z, Salisbury S, Moran NA. 2018. Pathogenicity of *Serratia marcescens* strains in honey bees. mBio, 9:e01649-18. https://doi.org/10.1128/mBio.01649-18
